# A New Compact Triple-Band Triangular Patch Antenna for RF Energy Harvesting Applications in IoT Devices

**DOI:** 10.3390/s22208009

**Published:** 2022-10-20

**Authors:** Chemseddine Benkalfate, Achour Ouslimani, Abed-Elhak Kasbari, Mohammed Feham

**Affiliations:** 1 Quartz Laboratory, Department of Electrical and Electronic Engineering, Ecole Nationale Supérieure de l’Electronique et de ses Applications, 95014 Cergy, France; 2 STIC Laboratory, Department of Telecommunications, Faculty of Technology, University Abou Bekr Belkaid, Tlemcen BP 230 13000, Algeria

**Keywords:** patch antenna, triple-band antenna, compact size, RF energy harvesting, IoT, antenna on waterproof paper, tilted antenna, rectifier, impedance matching

## Abstract

This work proposes a new compact triple-band triangular patch antenna for RF energy harvesting applications in IoT devices. It is realized on Teflon glass substrate with a thickness of 0.67 mm and a relative permittivity of 2.1. Four versions of this antenna have been designed and realized with inclinations of 0°, 30°, 60° and 90° to study the impact of the tilting on their characteristics (S_11_ parameter, radiation pattern, gain) and to explore the possibilities of their implementation in the architectures of electronic equipment according to the available space. The antenna is also realized on waterproof paper with a thickness of 0.1 mm and a relative permittivity of 1.4 for biomedical domain. All the antennas (vertical antenna, tilted antennas and antenna realized on waterproof paper) have a size of 39 × 9 mm^2^ and cover the 2.45 GHz and 5.2 GHz Wi-Fi bands and the 8.2 GHz band. A good agreement is obtained between measured and simulated results. Radiation patterns show that all the antennas are omnidirectional for 2.45 GHz and pseudo-omnidirectional for 5.2 GHz and 8.2 GHz with maximum measured gains of 2.6 dBi, 4.55 dBi and 6 dBi, respectively. The maximum measured radiation efficiencies for the three antenna configurations are, respectively, of 75%, 70% and 72%. The Specific Absorption Rate (SAR) for the antenna bound on the human body is of 1.1 W/kg, 0.71 W/kg and 0.45 W/kg, respectively, for the three frequencies 2.45 GHz, 5.2 GHz and 8.2 GHz. All these antennas are then applied to realize RF energy harvesting systems. These systems are designed, realized and tested for the frequency 2.45 GHz, −20 dBm input power and 2 kΩ resistance load. The maximum measured output DC power is of 7.68 µW with a maximum RF-to-DC conversion efficiency of 77%.

## 1. Introduction

**Figure 1:**
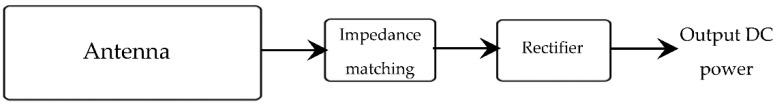
Blocs schematic of RF energy harvesting system.

The RF energy harvesting systems (RF-EH) consist of an antenna, impedance matching circuit and a rectifier, as shown in Figure 1 [1].

**Figure 2:**
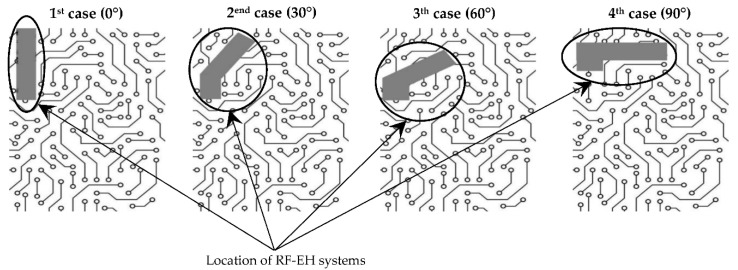
Location of the RF-EH systems in the four examples (0°, 30°, 60° and 90°) of equipment architectures to be supplied.

The size of these systems is defined mainly by the size of the used antenna, as the impedance matching circuit and the rectifier are designed on the basis of millimetric or even micrometric electrical elements [2,3,4,5]. The miniaturization of these RF-EH systems by reducing the size of the used antenna is recommended to facilitate their implementation in different architectures of embedded equipment to be fed [6]. In some cases, the implementation of a vertical antenna is possible, while in other cases it is impossible. The forms of sets allowing the implementation of these RF energy harvesting systems treated in this paper can be summarized in four cases as illustrated in Figure 2.

To overcome this problem, we study the effect of the tilt of this antenna of 30°, 60° and 90° from the vertical on its characteristics (S_11_ parameter and radiation pattern). The vertical antenna (0°) is considered as a reference.

RF energy harvesting systems are increasingly adopted by the biomedical field [7,8,9,10]. The application of RF energy harvesting in this field requires a minimum of flexibility of the antenna to allow a wide possibility of use on the human body. To meet these requirements, the antenna is realized on waterproof paper. It is simulated and tested for different types of deformation and for the case that it is glued on the human body in order to verify the stability of its characteristics.

The vertical antenna (0°) as well as the tilted antennas (30°, 60° and 90°) are realized on Teflon glass substrate with a thickness of 0.67 mm and a relative permittivity of 2.1. The flexible antenna is realized on waterproof paper with a thickness of 0.1 mm and a relative permittivity of 1.4. The proposed antenna (vertical antenna, tilted antennas and antenna realized on waterproof paper) has a size of 39 × 9 mm^2^.

Different antenna structures have been proposed in the literature for RF-EH applications [11,12,13,14,15,16,17,18,19,20,21,22,23,24,25,26,27,28]. The problem that often persists is the strong degradation of the gain once the antenna is slightly miniaturized. To meet this compromise, we propose in this paper a new compact triangular triple-band patch antenna structure that radiates at 2.45 GHz, 5.2 GHz and 8.2 GHz with a measured gain values of 2.6 dB, 4.55 dB and 6 dB for the three frequencies, respectively. 

The frequency 2.45 GHz allows exploitation of electromagnetic waves from Wi-Fi stations for indoor RF energy harvesting applications and electromagnetic waves from 4G(LTE) mobile network base stations for outdoor RF energy harvesting applications.

The frequency 5.2 GHz allow the system to exploit electromagnetic waves from WiFi stations for indoor RF energy harvesting applications and electromagnetic waves coming from the relay antennas of 5G mobile networks for outdoor RF energy harvesting applications.

The frequency 8 GHz is adopted to exploit electromagnetic waves from satellites (c and x bands) for outdoor RF energy harvesting applications.

The choice of the triangular shape is justified by three essential points:

- The simplicity of defining, theoretically, the resonant frequencies of the antenna according to its geometric parameters based on theory of resonant cavities (see Section 2);
- The maximum gain is higher compared to rectangular and circular shapes for the same resonators size due to the reduced surface area at the end of the triangle which increases the electric field intensity, as shown in Section 2;
- The superposition of several triangular resonators results in a low resistance at the connection point between them, which reduces the ohmic losses compared to the other shapes.

Table 1 shows a comparison between the proposed antenna and other miniaturized antennas reported in the literature in terms of size, resonant frequencies, maximum gain and type of used substrates.

The theoretical study of this antenna is conducted considering the triangular patches as resonant cavities to determine the resonant frequencies as a function of the geometrical parameters of the antenna [29,30,31].

The three antenna configurations (vertical, tilted and realized on waterproof paper) are omnidirectional for 2.45 GHz and pseudo-omnidirectional for 5.2 GHz and 8.2 GHz, which is suitable for RF-EH applications. The antennas are simulated on the CST software, characterized and tested. RF energy harvesting systems are designed, realized and tested using the proposed antennas for the frequency 2.45 GHz and −20 dBm input power.

## 2. Antenna Design

**Figure 3:**
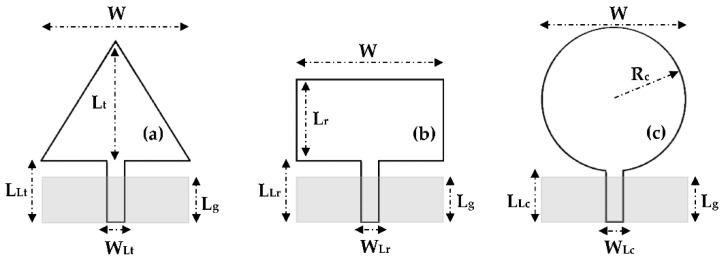
(**a**) Triangular, (**b**) rectangular and (**c**) circular antennas. 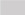: Bottom side.

To justify the choice of the triangular shape, three patch shapes (triangular, rectangular and circular) were simulated on Teflon glass substrate with 0.67 mm of thickness in terms of S_11_ parameters, electric and magnetic fields intensity for the frequency 5 GHz. The three resonators have the same area as presented in Figure 3. Their dimension values are given in Table 2. The dimensions of each resonator are calculated by using the equations given in [31,32,33] and then optimized on CST software. 

**Figure 4:**
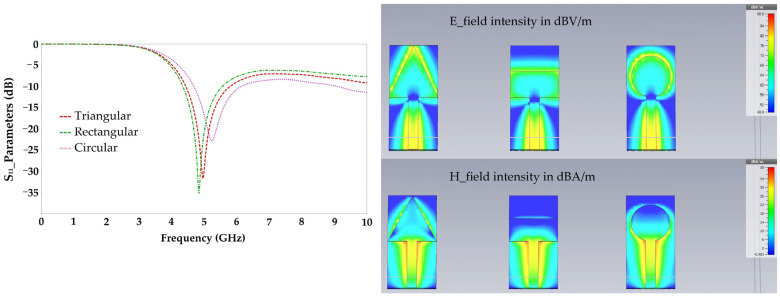
Simulated S_11_ parameters and (electric, magnetic) fields intensities for 5 GHz.

Figure 4 depicts the simulated S_11_ parameters, electric and magnetic fields intensities for each resonator shape.

Simulation results show that the triangular resonator has a relatively large E and H field intensity compared to that given by the circular resonator, especially at the end of the two resonators, and much larger than that given by the rectangular resonator. 

**Figure 5:**
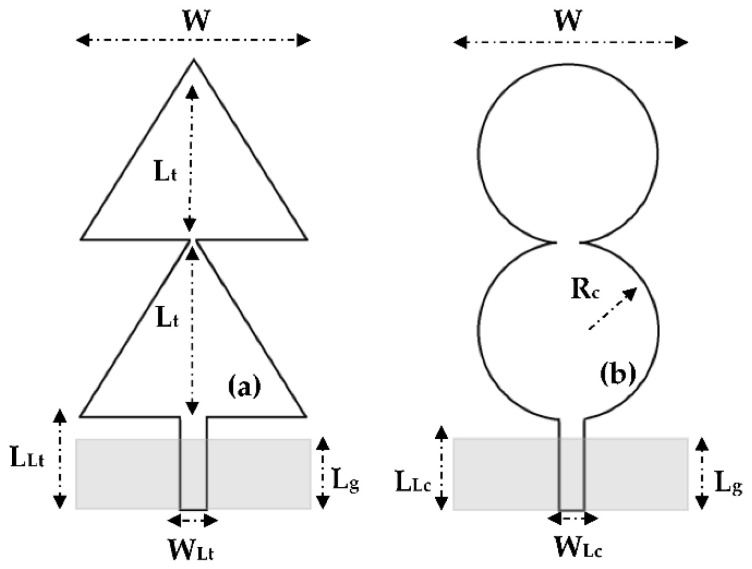
Two superposed resonators (**a**) Triangular and (**b**) circular antennas. 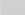: Bottom side.

For this reason, the study of the superposition of two resonators of the same area is focused on the triangular and circular resonators as presented in Figure 5.

**Figure 6:**
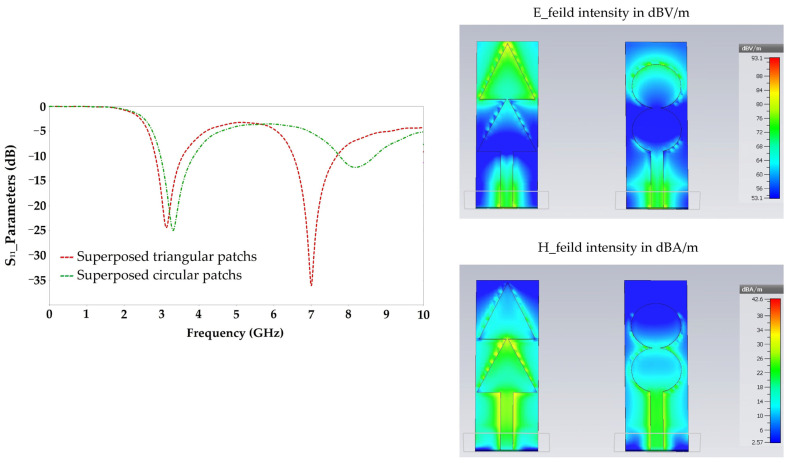
Simulated S_11_ parameters and (electric, magnetic) fields intensities for 3.3 GHz.

Figure 6 shows the simulation results in terms of S_11_ parameters, E and H field intensities of two superposed triangular and circular resonators for 3.3 GHz.

By analyzing the simulation results, it is clear that the triangular shape allows a good adaptation for two frequencies 3.3 GHz and 7 GHz, unlike the circular shape which is adapted only to the 3.3 GHz frequency. The E and H field intensities given by the triangular shape is much higher than that given by the circular shape. It is of 86 dBV/m and 33 dBA/m for the triangular shape and of 76 dBV/m and 26 dBA/m for the circular shape. A high E and H field intensities implies a high surface current density which gives a higher radiation efficiency. The simulated radiation efficiency for the 3.3 GHz frequency is of 82% for the triangular shape and of 70% for the circular shape.

All these results justify the choice of the triangular shape as an advantage over the rectangular and circular shapes for RF energy harvesting applications.

**Figure 7:**
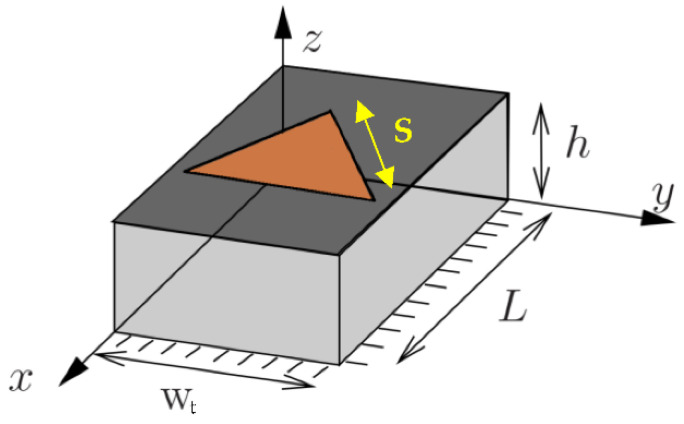
Triangular patch as resonant cavity.

The proposed antenna is formed by a superposition of three triangular patch resonators of the same size. The adopted feeding technique is suitable for exciting TM_m,n,p_ modes in the proposed antenna, whose resonant frequency can be calculated as function of antenna dimensions using the expression (1)–(4) given in [29,30,31] assuming that the patches behave as resonant cavities as presented in Figure 7.

(1) $$f_{r_{m,n,p}}=\frac{c_{0}}{2\pi \sqrt{\epsilon_{eff}}}\times \sqrt[{\;}]{{\left(\frac{4\pi }{3S}\right)}^{2}\left(m^{2}+mn+n^{2}\right)+{\left(\frac{p\pi }{2h_{eff}}\right)}^{2}}$$

where

(2) $$\epsilon_{eff}=\frac{\left(\epsilon_{r}+1\right)}{2}+\frac{\left(\epsilon_{r}-1\right)}{2}{\left(1+12\times \frac{W_{t}}{h}\right)}^{-\frac{1}{2}}\;\;\;\;$$

and

(3) $$h_{eff}=h\times \left(1-\epsilon_{r}{}^{-1}\right)$$

(4) $$f_{rOpt_{m,n,p}}\approx \frac{f_{r_{m,n,p}}}{2}$$

where *c*_0_ is the velocity of light in a vacuum and *h* the substrate thickness. *m*, *n* and *p* are TM propagation modes of the studied cavity (triangular patch), *S* is the side length of the triangle and $f_{rOpt_{m,n,p}}$ is the resonant frequency of the optimized antenna ground plan.

In our case, the dielectric is thin. The three fundamental modes are obtained for *p* = 0. These equations give a good theoretical approximation of the resonance frequencies for a full ground plane. The calculated dimensions are then optimized on the CST software for the purpose of miniaturization. For a ground plane length of less than 6 mm, the resonant frequency $f_{r_{m,n,p}}$ is reduced to half, which allows the miniaturization of the antenna dimensions. The proposed antenna structure excites the dominant TM_1,0,0_, TM_2,0,0_ and TM_3,0,0_ modes resulting on a triple-band antenna, which is not discussed in the available literature. This antenna is simulated and realized on the Teflon glass substrate with a relative electric permittivity of 2.1 and a thickness of 0.67 mm. The input impedance of this antenna is 50 Ω.

**Figure 8:**
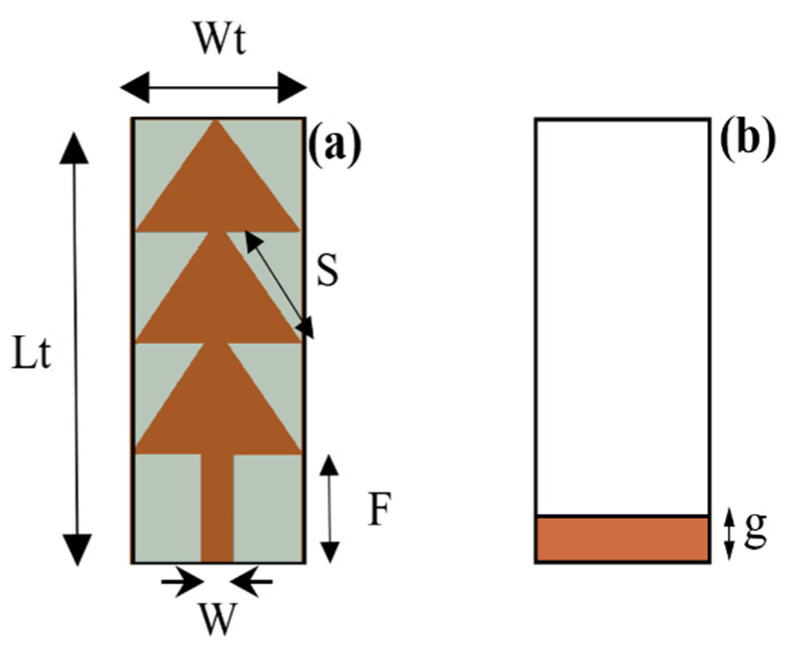
Proposed antenna shape. (**a**) Top side and (**b**) bottom side.

Figure 8 and Table 3 show the proposed antenna shape and it optimized dimensions, respectively.

**Figure 9:**
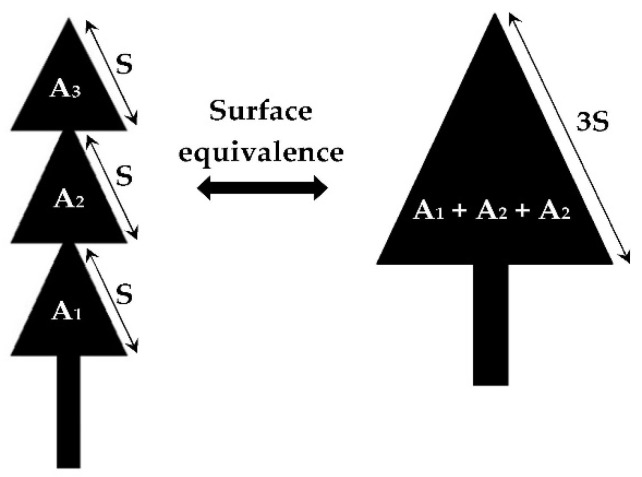
Equivalent patch area of the proposed antenna. A_i(i = 1, 2, 3)_ are the areas of each small triangular patch.

Figure 9 shows the area equivalence of the proposed antenna allowing to calculate resonant frequencies for the three fundamental modes TM_m,n,0_ as a function of the antenna dimensions. These resonant frequencies are then justified by simulation and measurement.

(5) $$f_{rOpt_{1,0,0}}=f_{rOpt_{0,1,0}}=\frac{c_{0}}{3\left(3S\right)\sqrt{\epsilon_{eff}}}=2.51\;GHz$$

(6) $$f_{rOpt_{2,0,0}}=f_{rOpt_{0,2,0}}=\frac{2c_{0}}{3\left(3S\right)\sqrt{\epsilon_{eff}}}=5.02\;GHz$$

(7) $$f_{rOpt_{3,0,0}}=f_{rOpt_{0,3,0}}=\frac{3c_{0}}{3\left(3S\right)\sqrt{\epsilon_{eff}}}=7.54\;GHz$$

**Figure 10:**
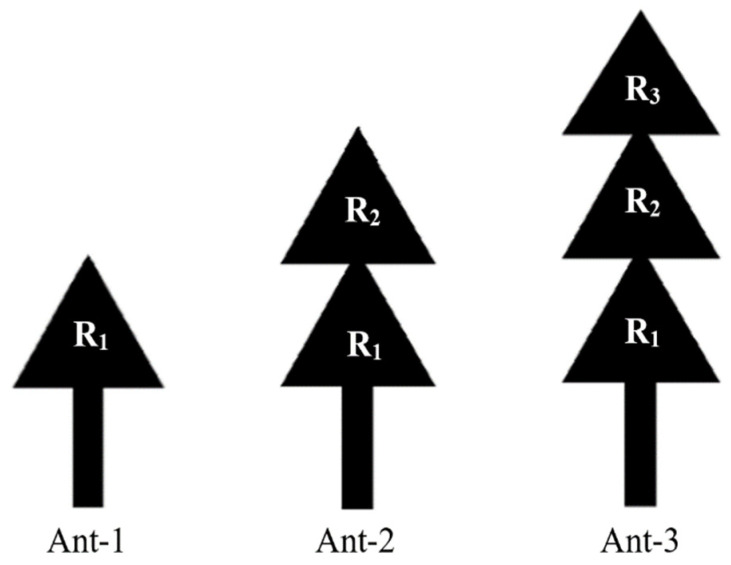
The studied antenna shapes.

To understand how this antenna radiates, we have simulated its response with one resonator, then with two and eventually with three resonators, as shown in Figure 10.

**Figure 11:**
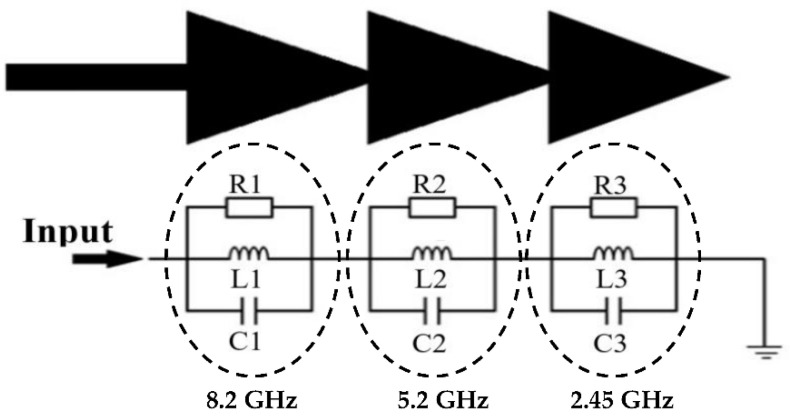
Electrical equivalent circuit of the proposed antenna.

The antenna electrical equivalent circuit is based on the antenna structure and the number of resonators. In our case, the proposed antenna consists of three triangular resonators, electrically equivalent to three cascaded RLC resonators [30], as shown in Figure 11.

The values of the elements R, L and C of the electrical equivalent circuit are calculated in Section 3 by exploiting the simulation results of the S_11_ parameter (bandwidths and resonant frequencies), the quality factor Q and the expression of the resonant frequency as a function of L and C for a parallel RLC resonator.

## 3. Simulation Results

**Figure 12:**
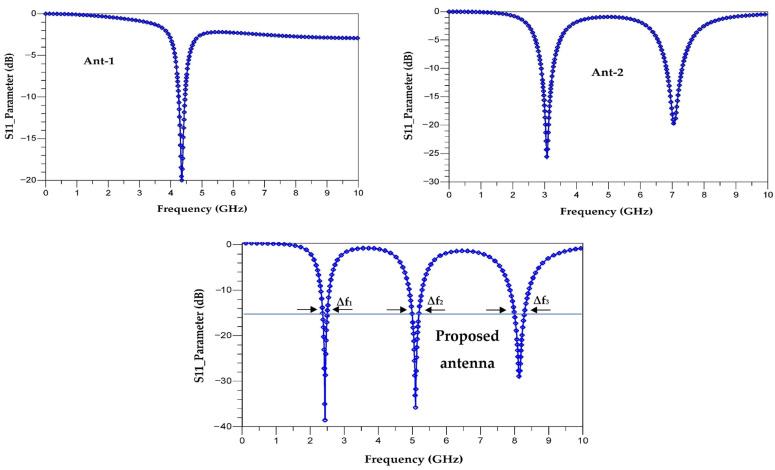
Simulated S_11_-parameters of the three antennas; ∆f_i(i = 1, 2, 3)_ are the frequency bandwidths.

Figure 12 shows the simulated S_11_ parameters of the antennas (Ant-1, Ant-2 and the proposed antenna (Ant-3)) under CST software.

The simulations show that the antenna (Ant-1) is matched to the resonance frequency of 4.4 GHz with an S_11_ of −20 dB, and the antenna (Ant-2) is adapted to the frequencies of 3 GHz and 7 GHz with reflection coefficients (S_11_) of −25 dB and −20 dB, respectively. The proposed antenna (Ant-3), is adapted to the three frequencies of 8.15 GHz, 5.2 GHz and 2.45 GHz with reflection coefficients S_11_ of −29 dB, −38 dB and −39.5 dB, respectively. The bandwidths for the frequencies 2.45 GHz, 5.2 GHz and 8.15 GHz covered by the proposed antenna are of ∆f_1_ = 6.6 MHz, ∆f_2_ = 20 MHz and ∆f_3_ = 32.16 MHz, respectively.

The quality factor Q of each RLC parallel resonator can be calculated for each frequency band (2.45 GHz, 5.2 GHz and 8.2 GHz) by Equation (8):

(8) $$Q=R\cdot \sqrt{\frac{L}{c}}=\frac{f_{r}}{\Delta f}$$

(9) $$f_{r}=\frac{1}{2\pi \sqrt{\mathrm{LC}}}$$

**Figure 13:**
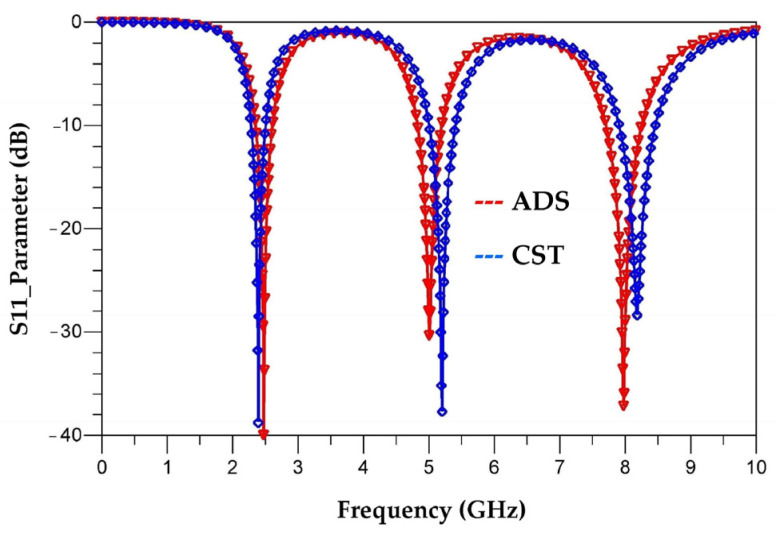
Simulated S_11_ parameters of the antenna and electrical equivalent circuit on CST and ADS software, respectively.

R, L and C are the resistance, inductance and capacitance of the resonators, respectively. f_r_ and Δf are the resonant frequency and bandwidth for S_11_ ≤ −15 dB as shown in Figure 13. Table 4 summarizes the values of Δf and Q for the three resonant frequencies.

From Equation (9), we fix one parameter (L) and deduce the other (C) for each resonance frequency. These values of inductance L, capacitance C and the passband for each resonant frequency are then used in Equation (8) to determine the values of the resistance R for each resonant frequency, i.e., 2.45 GHz, 5.2 GHz and 8.2 GHz.

Table 5 presents the values of RLC elements of the electrical equivalent circuit of the Figure 11.

Figure 13 shows a comparison between the simulated S_11_ parameter of the antenna done on CST and the simulated S_11_ parameter of the equivalent electrical circuit performed on ADS software.

It can be seen that the simulation of the equivalent electrical circuit response on ADS software and the electromagnetic (EM) simulation of the antenna response on CST are in good agreement. The electrical simulation shows a slight shift in the resonant frequencies to 8 GHz, 5 GHz and 2.5 GHz with reflection coefficients of −38 dB, −30 dB and −40 dB, respectively. It can be noted that the resonance frequencies simulated by the S_11_ parameter agree with the theoretical calculation made in Section 2 (Equations (5)–(7)).

**Figure 14:**
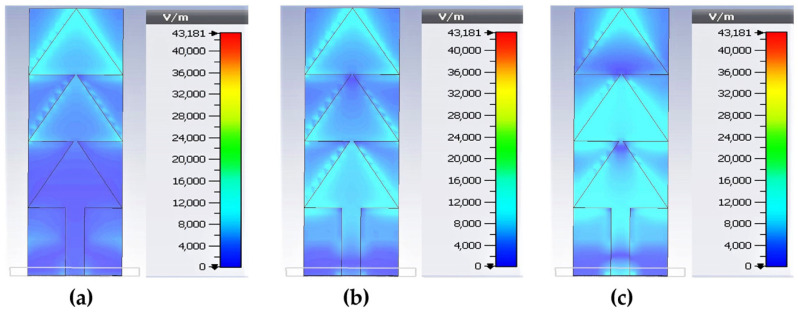
Comparison between E-field distribution of the antenna for (**a**) 2.45 GHz, (**b**) 5.2 GHz and (**c**) 8.2 GHz.

Figure 14 depicts the electrical field intensity distribution for 2.45 GHz, 5.2 GHz and 8.2 GHz.

For the frequency 2.45 GHz, we can see that the first maximum of the electrical (E) field intensity is located in the third triangle whose position corresponds to λg/4. For the frequency 5.2 GHz, the first maximum of the E-field intensity is located in the first triangle whose position corresponds to λg/4. The second maximum is located at the third triangle whose position corresponds to 3λg/4. For the 8.2 GHz frequency, the first maximum of the E-field intensity is located at 8 mm from the antenna excitation port corresponding to λg/4. The second maximum is located at the beginning of the second triangle whose position corresponds to 3λg/4. A third maximum occurs at the position 5λg/4.

**Figure 15:**
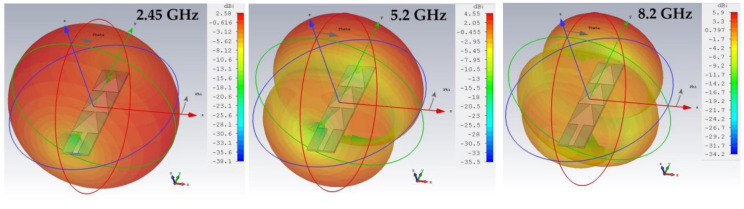
Simulated 3D radiation pattern of the proposed antenna for 2.45 GHz, 5.2 GHz and 8.2 GHz frequencies.

Figure 15 depicts the simulated 3D radiation pattern of the proposed antenna for 2.45 GHz, 5.2 GHz and 8.2 GHz, respectively.

It can be noted that the antenna is omnidirectional for the frequency 2.45 GHz with a maximum gain of 2.58 dBi. For the 5.2 GHz frequency, the antenna has two main lobes at the first and third triangle with a maximum gain of 4.55 dBi. For the 8.2 GHz frequency, the antenna has three lobes, the first is at 8 mm from the excitation port, the second is in the level of the second triangle and the third in the level of the last triangle with a maximum gain of 5.9 dBi. The position of the lobes corresponds to the position of the maximum intensity of the E field represented previously in Figure 14. The gain of an antenna is given as a function of the electrical field intensity by the Equation (10).

(10) $$G\left(\theta ,\;\varphi \right)=\frac{\epsilon_{\mathrm{ray}}\times 4\pi \times {\left|E\left(\theta ,\;\varphi \right)\right|}^{2}}{\int_{0}^{2\pi }\int_{0}^{\pi }{\left|E\left(\theta ,\;\varphi \right)\right|}^{2}\sin\left(\theta \right)d\theta d\varphi }$$

where $\epsilon_{\mathrm{ray}}$ is the radiation efficiency of the antenna, and θ and φ are the direction angles of far field radiation in polar plan.

It can be noted that the number of lobes represented by the antenna radiation pattern corresponds to the number of maxima of the E-field intensity. From Figure 14, the antenna at 2.45 GHz presents one maximum of E-field intensity, which is translated in Figure 15 by a single lobe forming an omnidirectional radiation pattern. Similarly, the antenna at 5.2 GHz has two maxima of E-field intensity which is represented by a radiation pattern with two-lobes in Figure 15. At 8.2 GHz, the antenna has three maxima of E-field intensity which implies a radiation pattern with three lobes as shown in Figure 15. The antenna is quasi-omnidirectional for 5.2 GHz and 8.2 GHz frequencies.

## 4. Measurement Results

**Figure 16:**
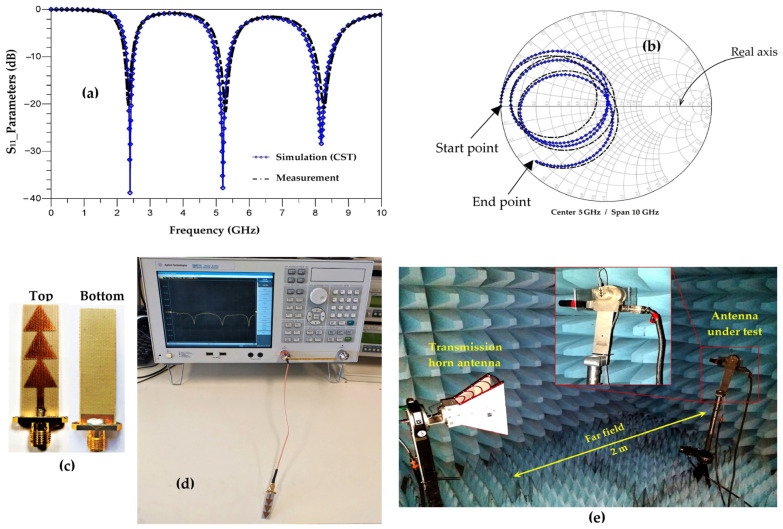
(**a**) Comparison between measured and simulated S_11_ parameters, (**b**) representation of S_11_ parameters on Smith chart, (**c**) realized antenna, (**d**) measurement prototype of S_11_ parameters and (**e**) measurement prototype of radiation pattern.

Figure 16, shows a comparison between the simulated and the measured S_11_ parameters of the proposed antenna (Ant-3) as well as the prototype of measurement.

Figure 16a shows that the measurement and simulation of the |S_11_|parameters are in good agreement. The three measured resonance frequencies, 2.45 GHz, 5.2 GHz and 8.2 GHz, correspond exactly to those simulated and calculated. The measured S_11_ parameters degrades a little compared to the simulated one, such that for the frequencies 2.45 GHz, 5.2 GHz and 8.2 GHz, the measured S_11_ parameter is of −20 dB, −23 dB and −20 dB, respectively. These measurement results justify the theoretical calculations and approximations of the resonant frequencies presented in Section 1.

From Figure 16b, we can see that the measured phase of S_11_ parameter concords with the simulated one. The points of intersection of the simulated S_11_ parameter curve with the real axis at the resonant frequencies 2.45 GHz, 5.2 GHz et 8.2 GHz are located on the 50 Ω impedance. The measurement shows a slight shift to the left and right of the 50 Ohm impedance. For the three resonant frequencies, the points of intersection of the measured S_11_ parameter curve with the real axis are located at the impedances of 45 Ω, 56 Ω and 57 Ω, respectively.

**Figure 17:**
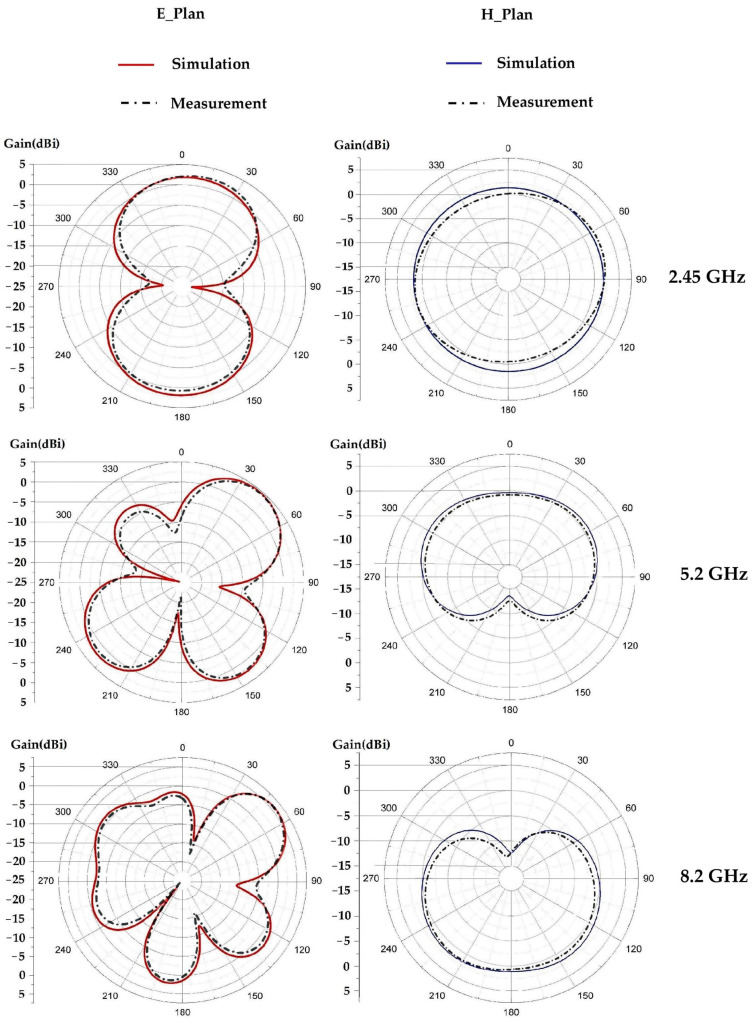
Measured 2D radiation pattern of the proposed antenna for 2.45 GHz, 5.2 GHz and 8.2 GHz frequencies.

Figure 17 depicts the simulated and measured radiation pattern of the proposed antenna compared to the simulated one for the frequencies 2.45 GHz, 5.2 GHz and 8.2 GHz. It can be noted that the measured and simulated radiation patterns are in good agreement. For the frequency 2.45 GHz the 3 dB-aperture for each lobe is of 170° in the E plane, which gives a radiation similar to a dipole antenna. For the 5.2 GHz frequency, the 3 dB-aperture for the main lobe is of 90°, and 70° for the two downward facing lobes and 30° for the left lobe. For the 8.2 GHz frequency, the 3 dB-aperture is of 110° for the left lobe, 60° for the right lobe and 30° for each downward facing lobe. For the three frequencies 2.45 GHz, 5.2 GHz and 8.2 GHz, the maximum measured gains are of 2.6 dBi, 4.55 dBi and 6 dBi, respectively. The simulated radiation efficiencies are of 74%, 72% and 66% for the frequencies 2.45 GHz, 5.2 GHz and 8.2 GHz, respectively. The measured radiation efficiencies are of 75%, 70% and 67% for the resonant frequencies 2.45 GHz, 5.2 GHz and 8.2 GHz, respectively.

## 5. Testing the Different Challenges That the Proposed Antenna Faces for RF Energy Harvesting Applications in IoT Devices

RF Energy Harvesting (RF-EH) applications are increasingly adopted to ensure the energy autonomy of low power consumption electronic equipment such as sensors, smart watches, biomedical equipment, etc. Two major obstacles that this antenna faces for this type of application can be pointed out. 

The first one is the architecture’s diversity of the electronic equipment to be powered, which are more and more complex. This poses a huge problem during the implementation in terms of antenna tilt. In some cases, the implementation of a vertical antenna is possible, while in other cases it is impossible, which requires the study of different tilting angles effects to solve this problem and make the RF-EH systems implementable regardless of equipment structures to be powered. The second one is imposed by the RF energy harvesting applications in the biomedical domain where the flexibility of the equipment is more and more recommended to give a large possibility of use (stick the equipment on the human body, facilitate the portability, … etc.). 

In this section the effects of tilt and flexibility on the response of the proposed antenna are simulated and tested.

### 5.1. Antenna Tilting Effects

**Figure 18:**
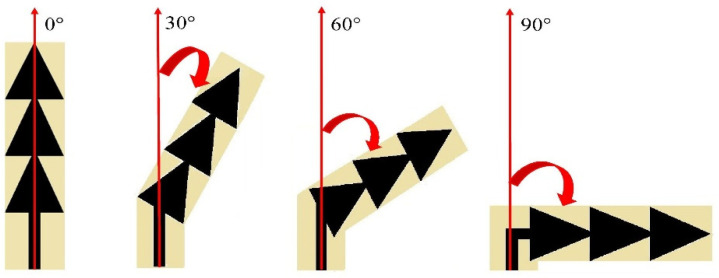
Different antenna tilting angles for RF-EH systems implementation.

**Figure 19:**
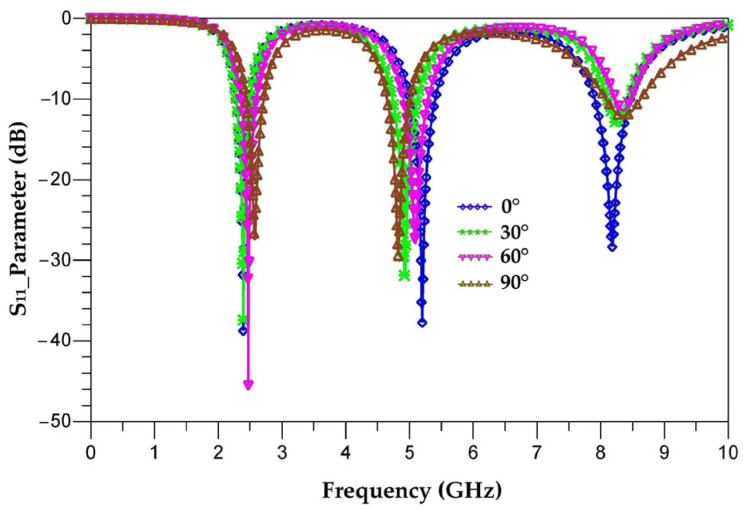
Simulated S_11_ parameters of tilted antennas (30°, 60° and 90°).

The aim of this part is to analyze the response of the proposed antenna for different tilting angles (30°, 60° and 90°) and to study the stability of its characteristics in order to avoid the efficiency degradation of RF-EH systems when they are implemented in electronic equipment. The tilted antennas are designed and realized on Teflon glass substrate with a thickness of 0.67 mm. Figure 18 depicts different antenna tilting shapes for 0°, 30°, 60° and 90° angles. Figure 19 presents the simulated S_11_ parameter of vertical antenna (0°) as well as of all of the tilted antennas (30°, 60° and 90°) to make a comparison and show the tilting effects.

It can be seen that the tilt of this antenna slightly affects its response. The non-tilted antenna covers the frequencies 2.45 GHz, 5.2 GHz and 8.2 GHz, where the S_11_ is equal to −39.5 dB, −38 dB and −29 dB, respectively. By tilting the antenna 30° from its vertical, the S_11_ parameter becomes −38 dB, −32 dB and −12.5 dB for the frequencies 2.45 GHz, 5 GHz and 8.4 GHz, respectively. At 60°, the S_11_ is equal to −48 dB, −27 dB and −12 dB for the frequencies 2.5 GHz, 5.1 GHz and 8.4 GHz, respectively. By tilting the antenna 90° from its vertical, the S_11_ drops to −27 dB, −28 dB and −12.5 dB for the frequencies 2.55 GHz, 4.95 GHz and 8.4 GHz, respectively. For these tilting angles, the antenna response changes slightly and covers the frequencies 2.45 GHz, 5.2 GHz and 8.2 GHz.

**Figure 20:**
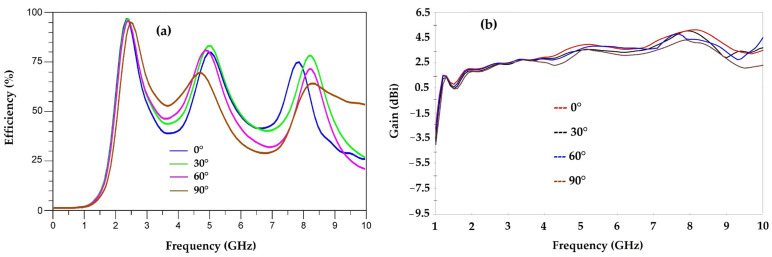
Antenna efficiency (**a**) and gain (**b**) as a function of frequency for the vertical antenna (0°) and the tilted antennas (30°, 60° and 90°).

Figure 20 shows the radiation efficiency and gain as a function of frequency for each tilted antenna.

It is seen that the efficiency of the antenna varies with its tilting angles. For 2.45 GHz, the efficiencies are of 96%, 95%, 92.5% and 92% for the angles 0°, 30°, 60° and 90°, respectively. For this frequency, the efficiencies are almost the same. For 5.2 GHz, the efficiencies are of 77.8%, 81%, 76% and 60% for the angles 0°, 30°, 60° and 90°, respectively. For this frequency, the maximum efficiency is obtained for the angle of 30° and degrades for the other angles of tilting. For the 8.2 GHz frequency, the efficiencies are of 72%, 68%, 60% and 58% for the angles 0°, 30°, 60° and 90°, respectively. The maximum efficiency is obtained for the vertical antenna (0°). We can note that the efficiencies remain above 58%.

We can note that for the 2.45 GHz frequency, the radiation pattern remains the same for the three tilt angles (30°, 60° and 90°) with a maximum gain of 2.44 dBi. For the 5.2 GHz frequency, we can see that the radiation pattern presents a little variation depending on the tilt angle, it becomes omnidirectional for the 90° angle. The maximum gain for the three tilt angles at this frequency is of 4.41 dBi. For the 8.2 GHz frequency, the radiation pattern changes shape slightly with a maximum gain of 5.47 dBi.

**Figure 21:**
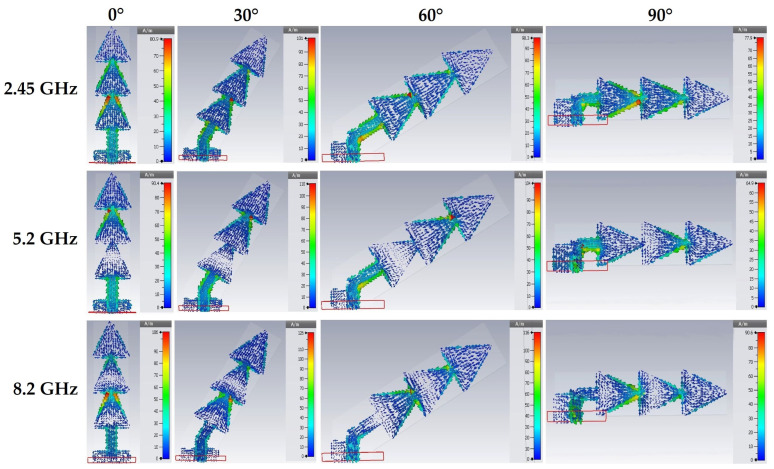
Simulated surface current density distribution for 2.45 GHz, 5.2 GHz and 8.2 GHz frequencies.

Figure 21 shows the variation of the surface current density for each antenna (0°, 30°, 60° and 90°) for the three resonant frequencies 2.45 GHz, 5.2 GHz and 8.2 GHz. 

For the three resonant frequencies 2.45 GHz, 5.2 GHz and 8.2 GHz, the surface current density is maximum for the 30° tilt angle. It reaches 101 A/m, 110 A/m and 126 A/m, respectively, for the three resonant frequencies. The surface current density is minimal for the 90° tilt angle. It is of 77.9 A/m, 64.9 A/m and 90.6 A/m, respectively, for the three resonance frequencies.

**Figure 22:**
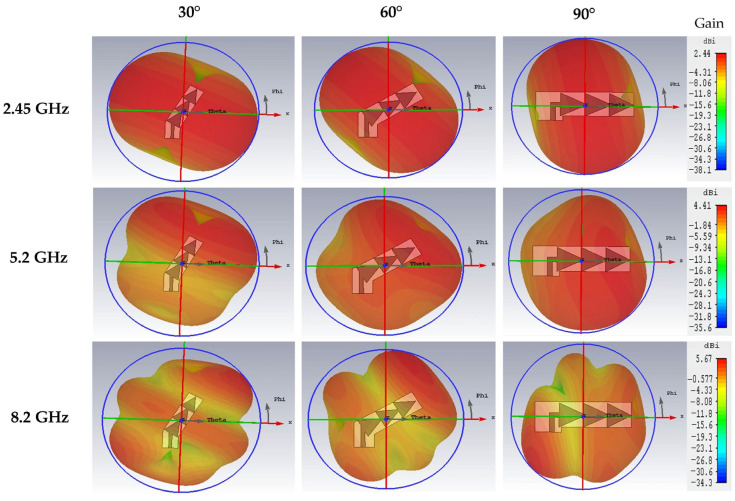
Simulated 3D radiation patterns of the three tilted antennas for 2.45 GHz, 5.2 GHz and 8.2 GHz frequencies.

Figure 22 shows the simulated 3D radiation pattern of the three tilted antennas (30°, 60° and 90°) for the three resonant frequencies 2.45 GHz, 5.2 GHz and 8.2 GHz.

**Figure 23:**
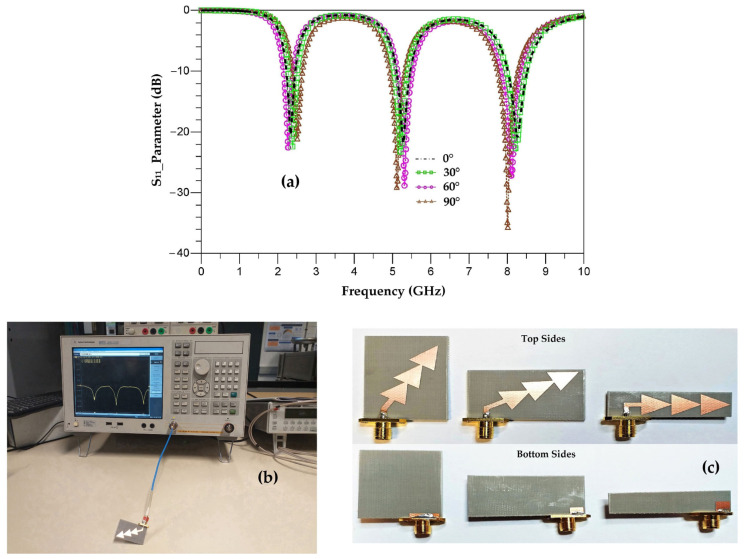
(**a**) Measured S_11_ parameters of the vertical antenna and the tilted antennas, (**b**) measurements prototype and (**c**) realized antennas.

Figure 23 compares the measured S_11_ parameters for each tilted antenna (30°, 60° and 90°) to that of the vertical antenna (0°), realized antennas and measurement prototype.

The measured and simulated S_11_ parameters are in good agreement for the two resonance frequencies 2.45 GHz and 5.2 GHz. The simulation (Figure 21) shows that the third resonance frequency is centered at 8.4 GHz for all three tilt angles (30°, 60° and 90°) while the measurement shows three different resonance frequencies of 8.2 GHz, 8.1 GHz and 8 GHz, respectively, for the three angles with improved S_11_ parameters. This difference between simulation and measurement for the frequency 8.2 GHz is explained in Section 8.

**Figure 24:**
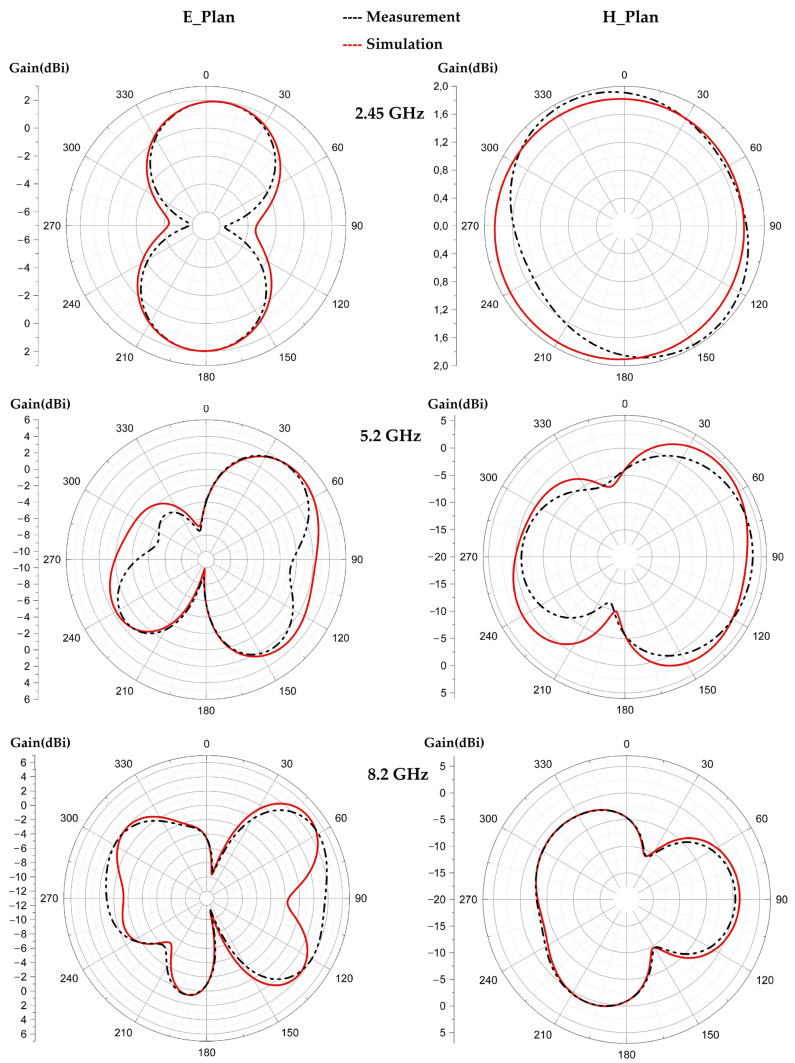
Measured and simulated radiation patterns (gain) of the 30° tilted antenna for the frequencies 2.45 GHz, 5.2 GHz and 8.2 GHz.

**Figure 25:**
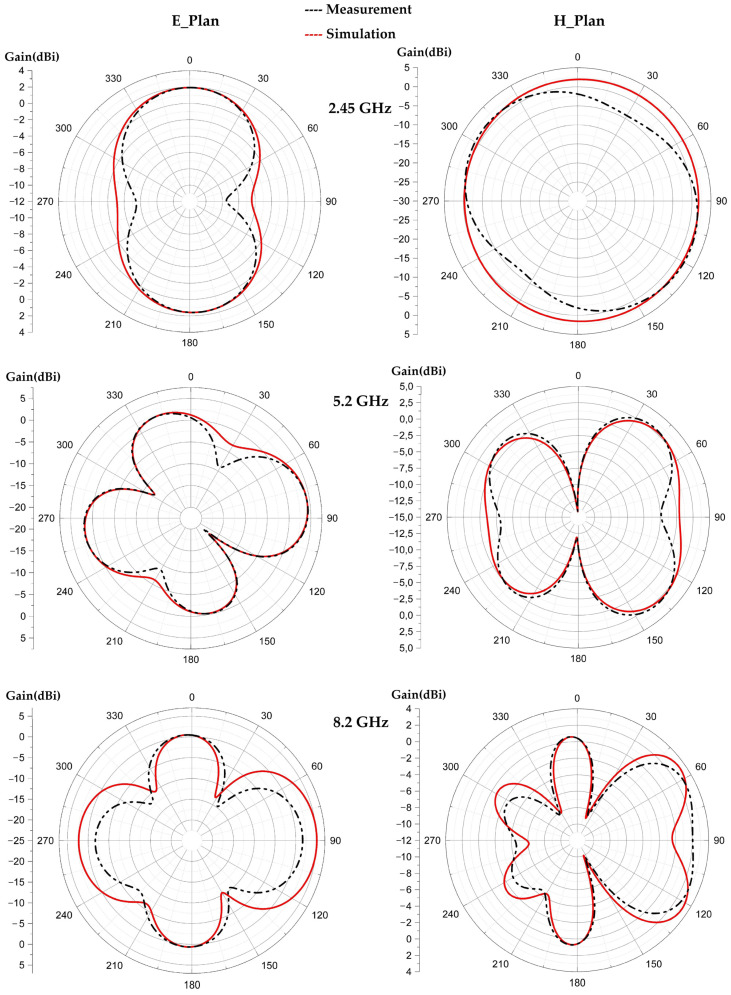
Measured and simulated radiation patterns (gain) of the 60° tilted antenna for the frequencies 2.45 GHz, 5.2 GHz and 8.2 GHz.

**Figure 26:**
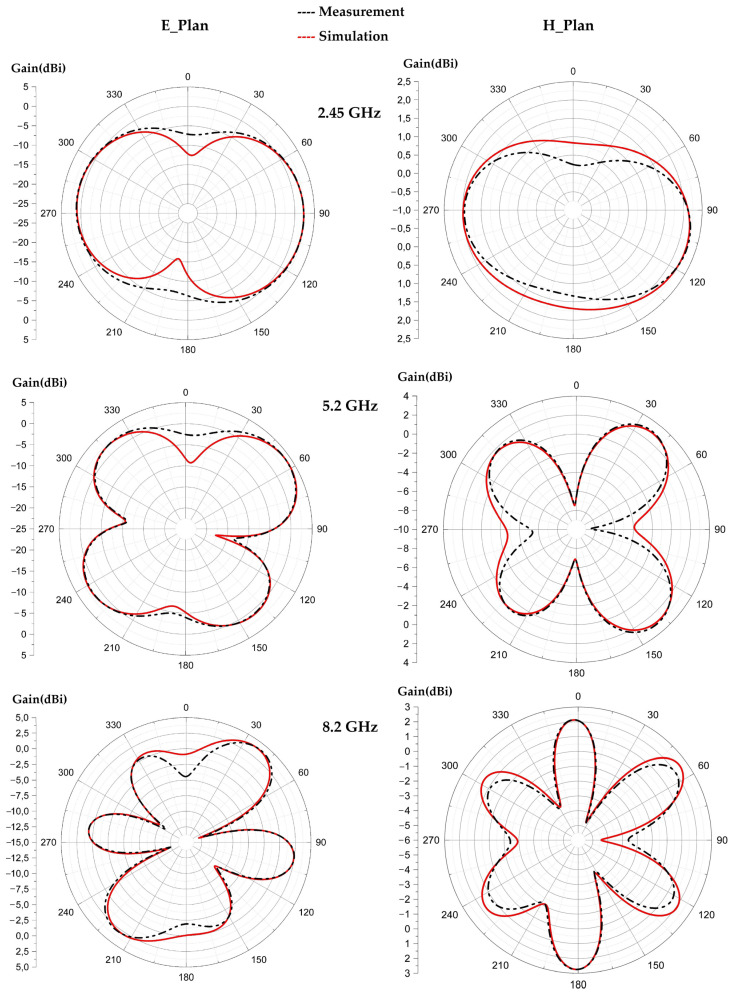
Measured and simulated radiation patterns (gain) of the 90° tilted antenna for the frequencies 2.45 GHz, 5.2 GHz and 8.2 GHz.

Figure 24, Figure 25 and Figure 26 show the simulated and measured 2D radiation patterns of each tilted antenna for the three resonant frequencies, 2.45 GHz, 5.2 GHz and 8.2 GHz, taking into account the coupling capacitor formed between the feed line and the first triangle of the antenna (see Section 8). The radiation patterns of the tilted antenna remain almost the same as those of the vertical one. For the 2.45 GHz frequency, a maximum gain of 2.44 dBi is given by the 90° tilted antenna. For the 5.2 GHz frequency, a maximum gain of 4.75 dBi is given by the antenna tilted by 60°. For the 8.2 GHz frequency, a maximum gain of 5.87 dBi is given by the antenna tilted by 30°. The shape of the pattern is almost the same from one tilt angle to another. The maximum measured efficiencies of the tilted antennas are of 75% for each one at 2.45 GHz; 70%, 72% and 68% for the tilted antennas of 30°, 60° and 90°, respectively, for 5.2 GHz; and 63%, 55% and 58% for the tilted antennas of 30°, 60° and 90°, respectively, for 8.2 GHz.

### 5.2. Antenna Flexibility for RF Energy Harvesting in the Biomedical Field

**Figure 27:**
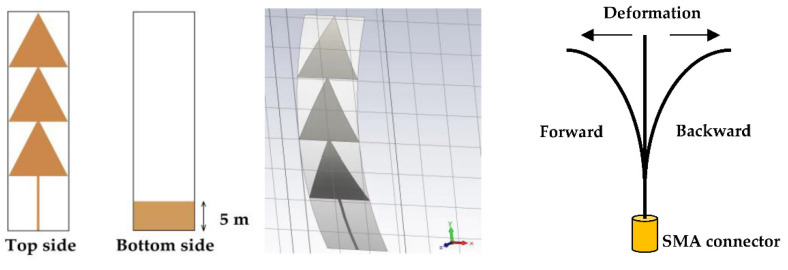
Designed antenna on waterproof paper.

The RF energy harvesting applications are increasingly exploited in the field of biomedicine in order to ensure the energy autonomy of medical monitoring equipment stuck on the human body. In this section, the proposed antenna is simulated and tested on waterproof paper with relative permittivity of 1.4 and 0.1 mm of thickness. Different antenna configurations are than tested (undeformed, deformed and glued on the human body). The width of the antenna feed line is 0.4 mm, which maintains a characteristic impedance of 50 Ω; all other dimensions remain unchanged as presented in Figure 27.

**Figure 28:**
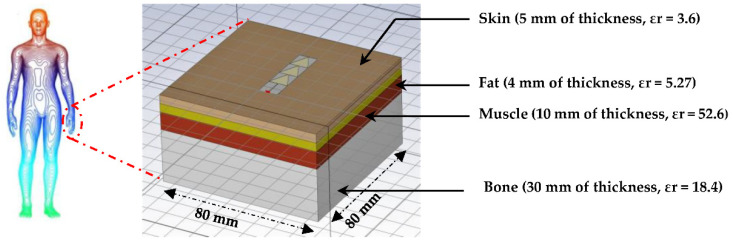
Designed waterproof paper antenna on human body.

To study the response of the antenna when bonded on the human body, we use the body model (hand) of [32,33,34,35] to perform this simulation as shown in Figure 28.

**Figure 29:**
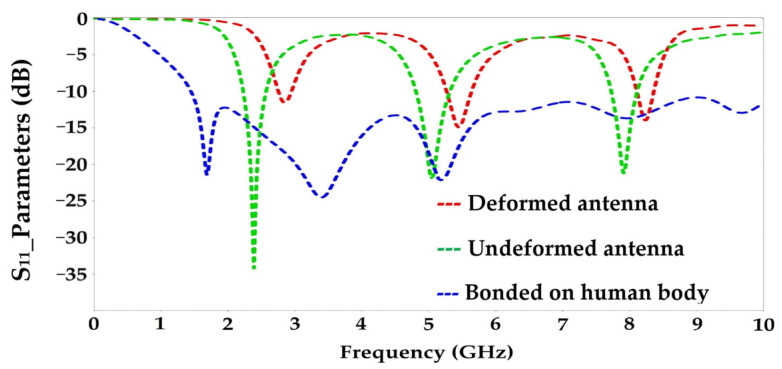
Simulated S_11_ parameters of the designed antenna on waterproof paper (undeformed and deformed cases) and bonded on human body.

Figure 29 shows the simulated S_11_ parameters of the undeformed, the deformed antennas and of the bonded antenna on the human body.

The designed antenna on waterproof paper is adapted to the three frequencies 2.45 GHz, 5.1 GHz and 8 GHz with S_11_ parameter magnitudes of −36 dB, −24 dB and −23 dB, respectively. The deformation of this antenna (forward or backward) shows some shifts of the resonant frequencies to 2.7 GHz, 5.5 GHz and 8.4 GHz, with relatively degraded S_11_ parameter magnitudes to −13 dB, −16 dB and −15 dB, respectively. For an S_11_ parameter of −10 dB, the deformed antenna covers the same frequencies as the undeformed one (2.5 GHz, 5.1 GHz and 8 GHz). 

It can be noticed that the human body affects the S_11_ parameter of the antenna by widening its bandwidth. It is of 8.5 GHz ranging from 1.5 GHz to 10 GHz. The antenna remains adapted for the three resonant frequencies 2.45 GHz, 5.2 GHz and 8.2 GHz.

**Figure 30:**
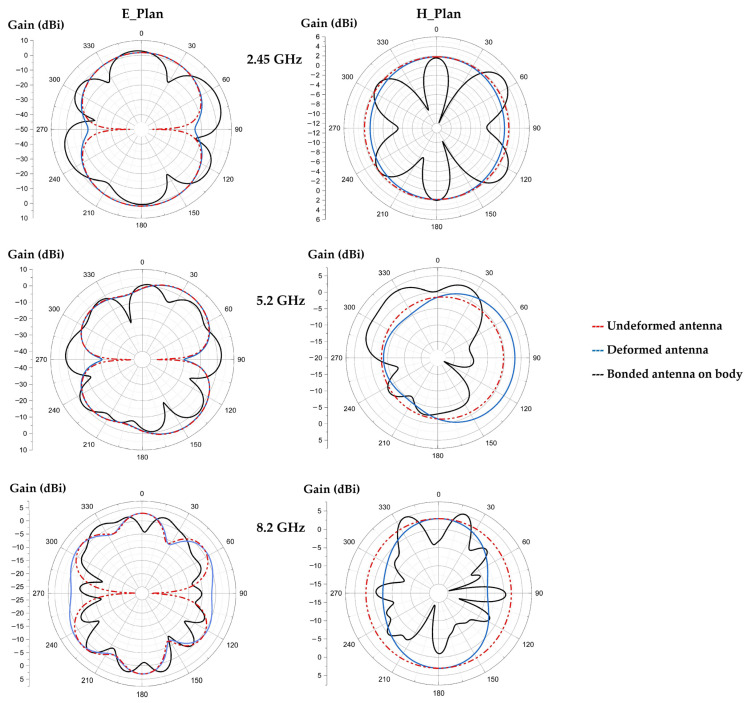
Simulated radiation patterns of the undeformed waterproof paper antenna, deformed one and of the bonded antenna on human body for the three resonant frequencies.

**Figure 31:**
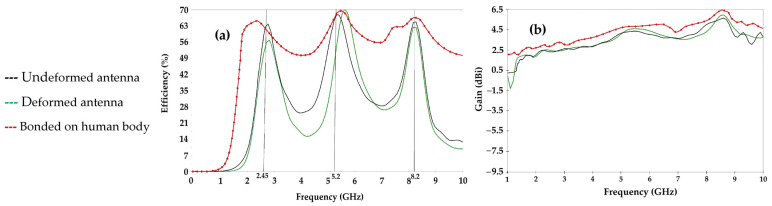
(**a**) Radiation efficiency, (**b**) gain as function of frequency for the undeformed antenna, deformed one and antenna glued on the human body.

Figure 30 shows the radiation patterns of the undeformed antenna, deformed one and of the antenna glued to the human body, for the three resonant frequencies. Figure 31 presents the simulated radiation efficiency and gain as function of frequency, respectively, for the three configurations. 

It can be noted that the radiation pattern of the antenna is affected a little by deforming it (forward or backward). The presence of the body changes the shape of the radiation pattern and allows to increase the gain values. 

For the frequencies 2.45 GHz, 5.2 GHz and 8.2 GHz, the simulated radiation efficiencies of the undeformed antenna are of 64%, 67% and 63% with gains of 2.4 dBi, 4.3 dBi and 4.8 dBi, respectively. The simulated radiation efficiencies of the deformed antenna are of 57%, 52% and 60% with gains of 2.42 dBi, 4.45 dBi and 4.8 dBi, respectively, for the three frequencies. The simulated radiation efficiencies of the bounded antenna on human body are of 61%, 66% and 65% with improved gains to 2.65 dBi, 4.67 dBi and 6.3 dBi, respectively, for the three resonant frequencies.

**Figure 32:**
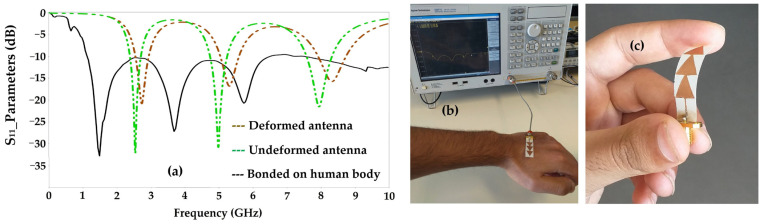
(**a**) Measured S_11_ parameters of the realized antenna on waterproof paper (undeformed, deformed and bonded on body cases), (**b**) measurement prototype and (**c**) realized antenna on waterproof paper (deformed).

Figure 32 shows the measured S_11_ parameters of the realized antenna on the waterproof paper, the deformed antenna and glued on the human body. The figure also shows the realized antenna and the measurement prototype.

The measurement and simulation results are in good agreement. The undeformed antenna covers the frequencies 2.45 GHz, 5 GHz and 8 GHz with S_11_ parameter amplitudes of −33 dB, −32 dB and −23 dB, respectively. The deformation of this antenna affects its response a little, such that the covered frequencies are of 2.6 GHz, 5.3 GHz and 8.3 GHz with a little degraded amplitudes of the S_11_ parameter to −22 dB, −18 dB and −16 dB, respectively. The human body broadens the bandwidth of the antenna, as shown in Figure 32. The measured bandwidth is of 8.8 GHz ranging from 1.2 GHz to 10 GHz for an amplitude of the S_11_ parameter less than or equal to −10 dB.

**Figure 33:**
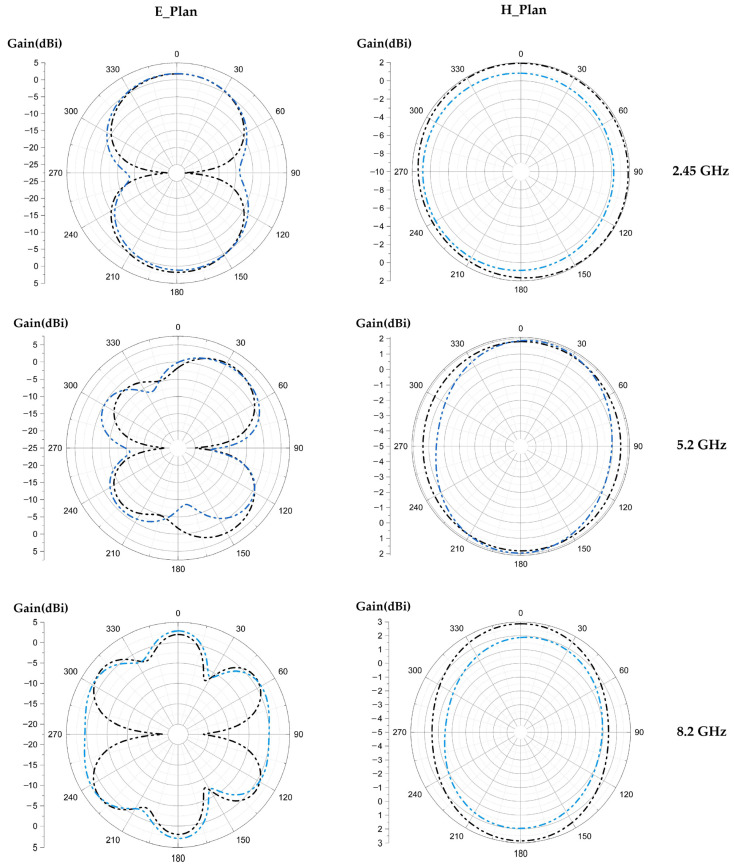
Measured radiation patterns of the undeformed waterproof paper antenna and deformed one for the three resonant frequencies 2.5 GHz, 5.2 GHz and 8.2 GHz.

Figure 33 shows the measured radiation patterns of the undeformed antenna and of the deformed antenna for the three resonant frequencies 2.5 GHz, 5.2 GHz and 8.2 GHz. 

The undeformed and deformed antennas are omnidirectional for 2.45 GHz and quasi-omnidirectional for 5.2 GHz and 8.2 GHz. The deformation of the waterproof paper antenna does not change the radiation pattern of the antenna much, as shown in Figure 33. The maximum measured gains for the undeformed antenna are 2.45 dBi, 4.3 dBi and 4.8 dBi, with maximum measured radiation efficiencies of 58%, 61% and 66%, for the frequencies 2.45 GHz, 5.2 GHz and 8.2 GHz, respectively. For the deformed antenna, the maximum gains are of 2.43 dBi, 4.4 dBi and 4.7 dBi, with maximum measured radiation efficiencies of 55%, 57.5% and 62% for the three resonant frequencies, respectively.

## 6. Specific Absorption Rate (SAR) Investigation

The study of the specific absorption rate of the undeformed and the deformed antenna bounded on the human body (arm) for the frequencies of 2.4 GHz, 5.2 GHz and 8.2 GHz is presented. Only the bending in the E-plane has been taken into account. The SAR analysis is performed with the CST software, using the IEEE C95.3 averaging method. SAR calculations were performed on 10 g of tissue volume (ICNIRP). The SAR standard safety level is 2 W/kg for 10 g of tissue [36].

The human body is a conductive and dielectric material. When the antenna is stuck to the human body, it is mismatched due to the impedance change and some of the available power is absorbed by the human body, resulting in higher Specific Absorption Rate (SAR) values.

The SAR can be calculated using the expression (11).

(11) $$\mathrm{SAR}=\frac{\sigma \left|E\right|2}{\rho }$$

where, σ is the tissue electrical conductivity, ρ is the tissue density (kg/m^3^) and E is the root-mean-square electric field induced in the tissue (V/m).

**Figure 34:**
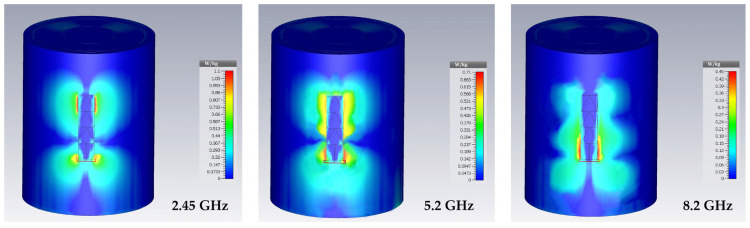
Specific absorption rate for 10 g of tissue and 100 mW input power at 2.5 GHz, 5.2 GHz and 8.2 GHz.

Figure 34 presents the SAR simulation of the flexible antenna bounded on human body (arm) for 10 g of tissue and 100 mW input power at 2.45 GHz, 5.2 GHz and 8.2 GHz. The arm is modelized in cylindrical form on CST software [37]. The input power of 100 mW is the maximum authorized for wearable applications [36].

It can be seen that the peaks of SAR_10g_ are of 1.1 W/kg, 0.71 W/kg and 0.45 W/kg for the three frequencies 2.45 GHz, 5.2 GHz and 8.2 GHz, respectively. The European standard of 2 W/kg is thus respected.

## 7. Design of the RF Energy Harvesting System Using the Proposed Antennas

In this part, the application of the antennas (vertical antenna, inclined antennas and antenna made on waterproof paper) presented in this paper to realize an RF energy harvesting system is tested. These systems are simulated, characterized and tested.

**Figure 35:**
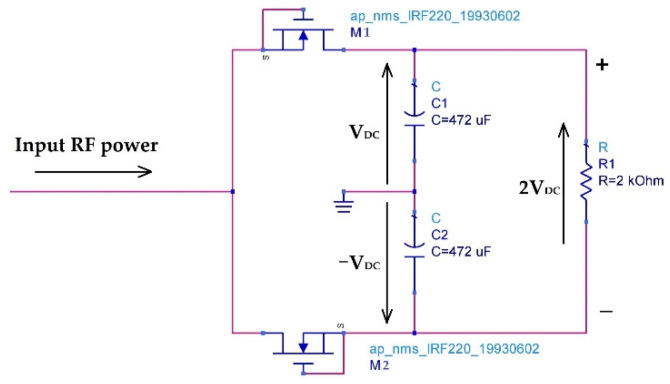
Designed rectifier on ADS software.

The rectifier circuit is realized based on two MOSFET transistors (IRF220) and two capacitors forming a DC-to-DC voltage doubler, as presented in Figure 35.

**Figure 36:**
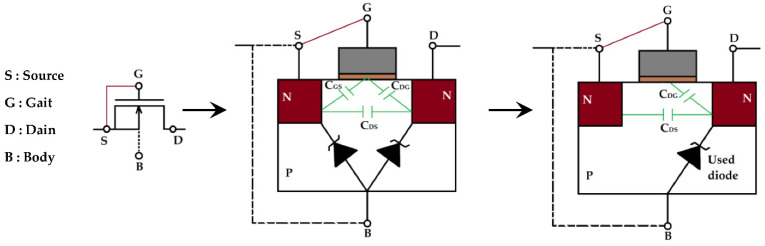
Representation of the exploited inner diode of the used nMOSFET transistor.

**Figure 37:**
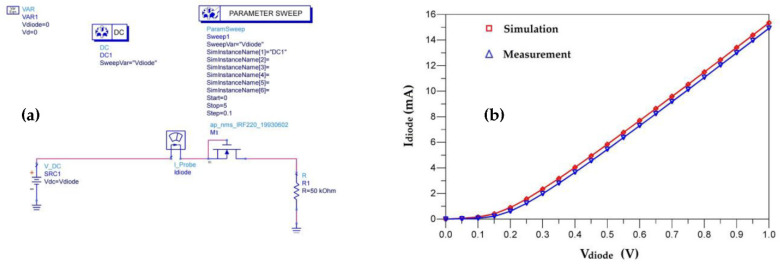
(**a**) Transistor characterization on ADS software and (**b**) simulated and measured characteristics of the intrinsic diode of the used nMOS transistor.

The transistors are used as a diode by connecting the source with the bulk as shown in Figure 36. The threshold voltage of this inner diode of the transistor is very low and it is of 0.17 V. Figure 37 shows the measured and simulated characteristic of this diode.

The choice of a 50 kΩ resistor allows analysis of the behavior of the transistor (intrinsic diode) for currents in the µA range. This corresponds to the average currents recovered by the RF energy harvesting systems for an input power lower than −5 dBm and a frequency of 2.45 GHz [38,39,40,41,42,43].

The internal capacitances of the MOSFET transistor (C_GD_ and C_DS_) increase the time constant τ of the rectifier. This allows an increase in the average of the output DC signal V_DC_. The time constant τ of the rectifier is given by the Equation (12).

(12) $$\tau =R_{L}C_{\mathrm{eq}}$$

(13) $$C_{\mathrm{eq}}=2(C+C_{\mathrm{GD}}+C_{\mathrm{DS}})$$

**Figure 38:**
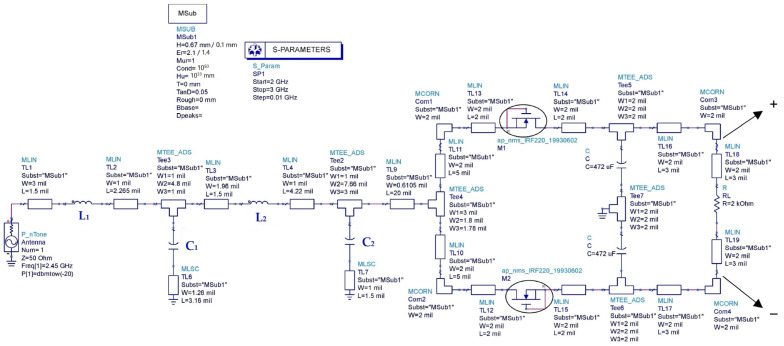
Designed matched rectifier for –20 dBm of input power, 2.45 GHz frequency and 2 kΩ resistance load.

To match the rectifier to the antenna with a characteristic impedance of 50 Ω for the 2.45 GHz frequency, an impedance matching circuit formed by a superposition of two LC resonators in L structure is proposed as shown in Figure 38 [44].

Table 6 shows the values of the LC elements of the impedance matching circuit for the two substrates Teflon glass with a relative permittivity of 2.1 and a thickness of 0.67 mm, and waterproof paper with a thickness of 0.1 mm and a relative permittivity of 1.4.

**Figure 39:**
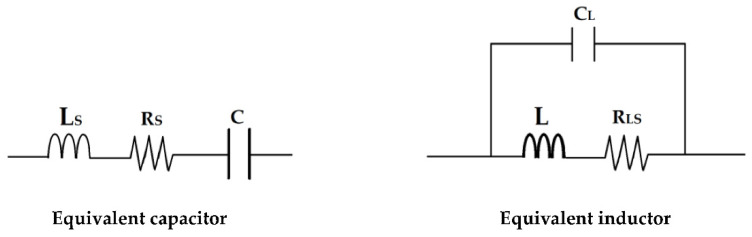
Equivalent capacitor and inductor circuit in microwave domain. L_s_, R_s_, R_LS_ and C_L_ are the series parasitic inductor and resistor of the selected capacitor, the parasitics resistor and capacitor of the selected inductor.

At the 2.45 GHz frequency, the capacitors and inductors present parasitic elements such that their equivalent structures are presented in Figure 39 [45,46].

The values of the parasitic parameters are determined using the expressions (14–17) of the quality factor Q and of the resonant frequency of a series and parallel RLC resonators, respectively. The value of the quality factor Q is provided by the manufacturer of the element (L and C) in their datasheet.

(14) $$Q_{\mathrm{Serie}}=\frac{1}{R_{S}}\cdot \sqrt{\frac{L_{S}}{C}}$$

(15) $$Q_{\mathrm{Parallel}}=R_{\mathrm{LS}}\cdot \sqrt{\frac{C_{L}}{L}}$$

(16) $$\omega_{\mathrm{Serie}}=\frac{1}{\sqrt{L_{S}C}}$$

(17) $$\omega_{\mathrm{Parallel}}=\frac{1}{\sqrt{{\mathrm{LC}}_{L}}}$$

**Figure 40:**
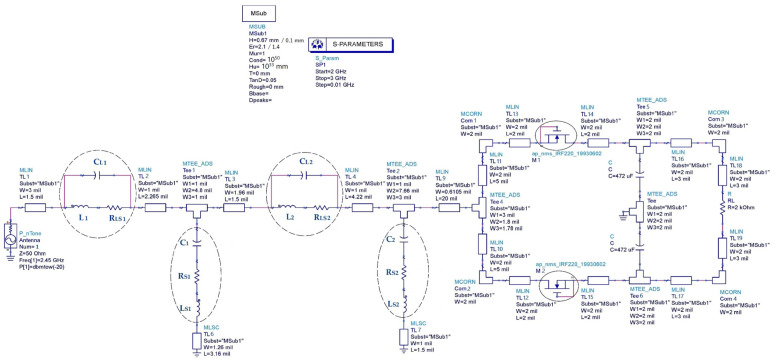
Designed matched rectifier with parasitic elements of the impedance matching circuit for –20 dBm of input power, 2.45 GHz frequency and 2 kΩ resistance load.

Figure 40 shows the rectifier circuit taking into account the parasitic elements of the impedance matching circuit. Table 7 gives the values of the parasitic elements for the two substrates Teflon glass and waterproof paper.

**Figure 41:**
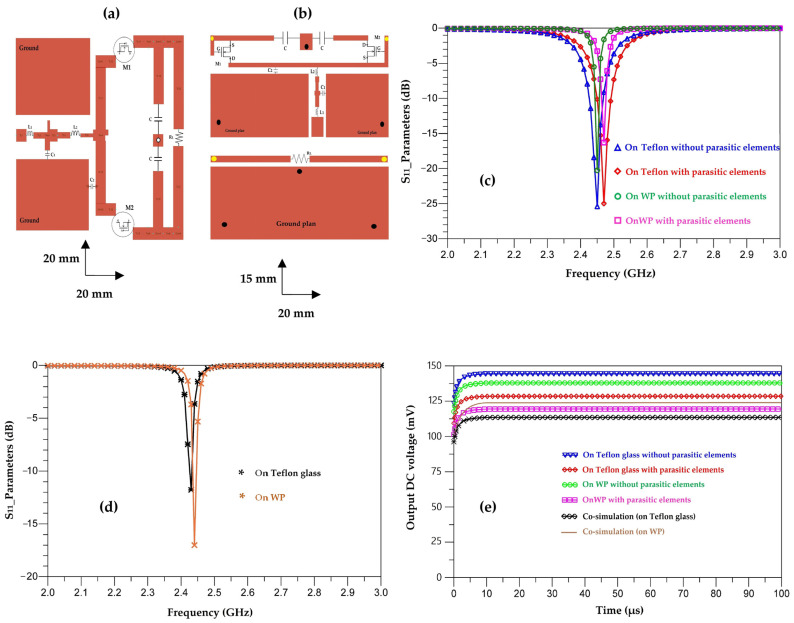
(**a**,**b**) Layout of the matched rectifiers on WP and Teflon glass substrates, respectively, with optimized dimensions, (**c**) simulated S_11_ parameters of the matched rectifiers with and without parasitic elements, (**d**) co-simulated S_11_ parameters and (**e**) simulated output DC voltages, all for −20 dBm input power, 2.45 GHz frequency and 2 kΩ resistance load.

Figure 41 depicts the layout of the matched rectifier with optimized dimensions on ADS software and the simulated S_11_ parameters of the matched rectifiers with and without parasitic elements for both used substrates (Teflon glass and waterproof paper (WP)).

It can be seen that the simulated output DC voltages are of 140 mV, 137 mV, 127 mV and 115 mV, for the designed rectifiers on Teflon glass substrate without and with parasitic elements, on waterproof paper without and with parasitic elements, respectively. 

The co-simulated output DC voltages are of 112 mV, 123 mV for the designed rectifiers on waterproof paper and Teflon glass substrates, respectively.

**Figure 42:**
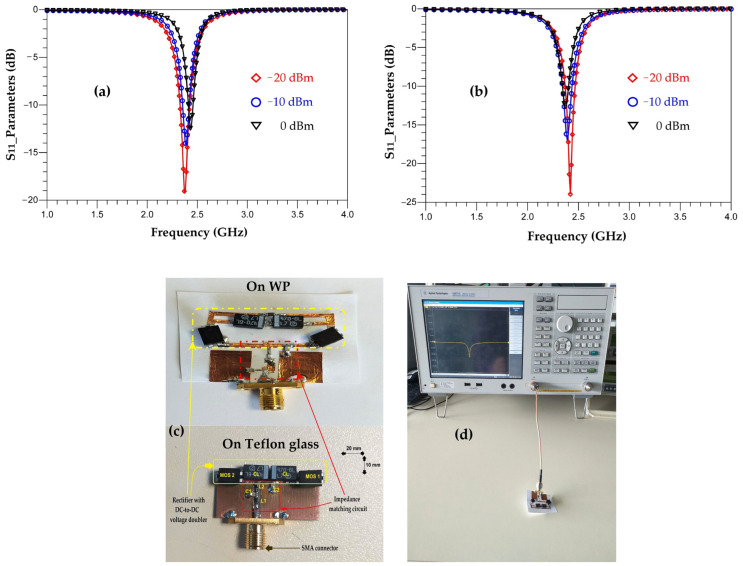
Measured S_11_ parameters of the rectifier realized on (**a**) Teflon glass; (**b**) WP for input powers of −20 dBm, −10 dBm and 0 dBm and 2 kΩ resistance load; (**c**) realized rectifiers; and (**d**) measurement prototype.

Figure 42 shows the realized rectifiers on Teflon glass and waterproof paper substrates and their measured S_11_ parameters for input powers of −20 dBm, −10 dBm and 0 dBm.

We can see that both rectifiers are matched to the 2.45 GHz frequency for the three input powers. For −20 dBm, the magnitude of the measured S_11_ parameter for the rectifier realized on Teflon glass substrate is of −19 dB and of −24 dB for the rectifier realized on WP substrate. As the input power increases, the amplitude of the S_11_ parameter decreases, such that for an input power of 0 dBm, the amplitude of the measured S_11_ parameter for the rectifier realized on Teflon glass substrate is of −15 dB and of −14 dB for the rectifier realized on WP substrate.

**Figure 43:**
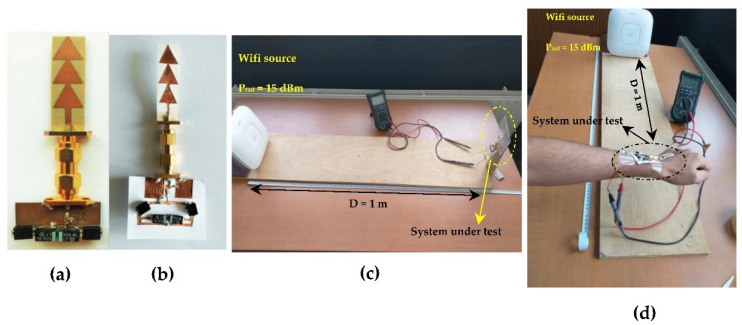
Realized RF energy harvesting systems on (**a**) Teflon glass, (**b**) WP substrate and (**c**,**d**) prototypes of measurement.

Figure 43 shows the prototype of the output DC voltage and RF-to-DC conversion efficiency measurements of the RF energy harvesting systems (RF-EH) using a commercial Wi-Fi source. 

Table 8 and Table 9 summarize the measured output DC voltages, the output DC powers and RF-to-DC conversion efficiencies for an input power of −20 dBm and 2 kΩ resistance load.

The RF-to-DC conversion efficiency is given by the expression (18)

(18) $$\eta =\frac{P_{\mathrm{out}}}{P_{\mathrm{int}}}=\frac{V_{\mathrm{DC}}^{2}}{R_{L}P_{\mathrm{int}}}$$

P_out_, P_int_, V_DC_ and R_L_ are the output DC power, the input power, the output DC voltage and the resistance load, respectively.

The variation of the output DC voltage and DC power from one configuration to another depends mainly on the antenna gains as well as the magnitudes of the S_11_ parameters of the antennas and rectifiers. The received RF power by the RF-EH system is given by the Friis Equation (19) [47].

(19) $$P_{r}=\left(1-{\left|\Gamma_{r}\right|}^{2}\right)\cdot G_{r}\cdot {\left(\frac{\lambda }{4\pi D}\right)}^{2}\cdot \left(1-{\left|\Gamma_{t}\right|}^{2}\right)\cdot G_{t}\cdot P_{t}$$

P_r_, P_t_, G_r_, G_t_, $\Gamma_{r}$, $\Gamma_{t}$, D and λ are the received power, the transmission power, the gain of the transmitting antenna, the gain of the receiving antenna, the coefficient reflection of the receiving antenna, transmitting antenna, distance between both antennas and the wavelength of the received RF signal. 

As can be seen, the measured output DC voltages remain fixed around an average DC voltage of 110 mV for −20 dBm input power and 2 kΩ resistance load. The output DC voltage provided by the system bonded to the human body is relatively high compared to the DC voltages provided by the other configurations; this is due to the increased gain when the antenna is bonded on the human body, as shown before in Figure 32.

Table 10 presents a comparison between the proposed RF-EH system and other systems reported in the literature in terms of size, output DC voltage, maximum RF-to-DC conversion efficiency and used substrate.

## 8. Discussion

**Figure 44:**
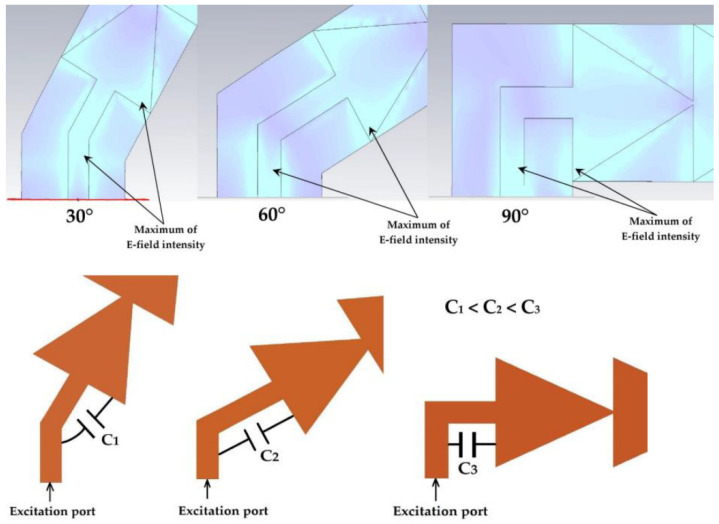
Equivalent capacitance for each tilting angle.

In Section 5.1, the simulation results of the S_11_ parameter represented in Figure 21 showed a difference with the measurement for the 8.2 GHz frequency of the three tilt angles, 30°, 60° and 90°. The simulation shows that the antennas are matched to the 8.4 GHz frequency with amplitudes of the S_11_ parameters of −13 dB. The bandwidth increases as a function of the tilt angle. The measurement showed that the antennas tilted by 30°, 60° and 90° are matched to the frequencies 8.2 GHz, 8.1 GHz and 8 GHz, respectively. The measured S_11_ parameter amplitudes are improved compared to the simulation. This can be explained by the fact that tilting the antenna results in the formation of an equivalent capacitance between the feed line and the first triangle Cc (between the first two maximums of the E-field intensities for 8.4 GHz frequency), as shown in Figure 44.

This capacitance improves the quality factor Q_f_ of the antenna at these frequencies (8.2 GHz, 8.1 GHz and 8 GHz), which explains the improvement of the measured S_11_ compared to the simulated one. The two simulated and measured resonance frequencies are given by the following formulas.

(20) $$F_{r,sim}=\frac{1}{2\pi \sqrt{LC}}$$

(21) $$F_{r,meas}=\frac{1}{2\pi \sqrt{L\left(C+C_{C}\right)}}$$

(22) $$C_{C}=C\left({\left(\frac{F_{r,sim}}{F_{r,meas}}\right)}^{2}-1\right)$$

Table 11 gives the values of the coupling capacitances for the three angles (30°, 60° and 90°). The capacitance *C* is the same as the capacitance *C*_1_ = 3.92 pF of the first RLC resonator of the electrical equivalent circuit corresponding to the frequency 8.4 GHz as seen in Section 2 (page 8).

**Figure 45:**
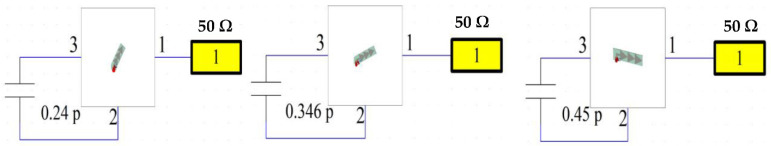
Simulation of the three tilted antennas taking into account the coupling capacitances.

In order to improve the simulation results on CST software, the capacitances *C_C_* are added between the first triangle and the feed line corresponding to the two maximums of the E field intensity points for each tilted antenna, as shown in Figure 45.

**Figure 46:**
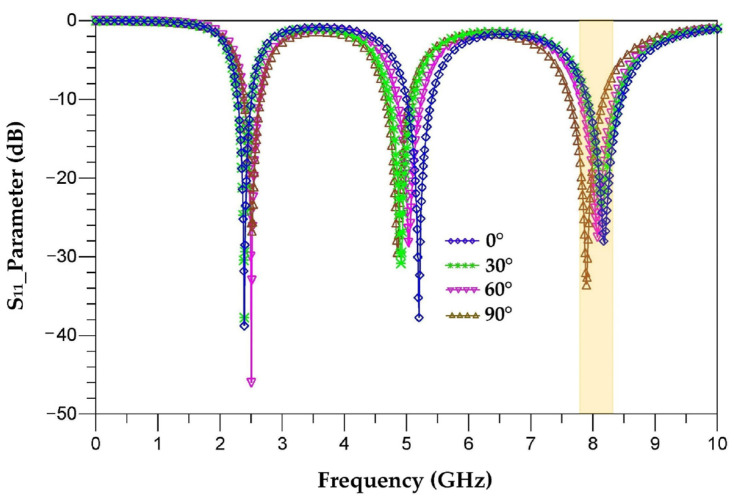
Simulated S_11_ parameters of the tilted antennas (30°, 60° and 90°) with coupling capacitances.

Figure 46 depicts the simulated S_11_ parameter of each configuration (30°, 60° and 90°). We can see that the new simulation taking into account the coupling capacitance is in good agreement with the measurement for the three resonance frequencies. The quality factor is given by the formula (23) as a function of *Cc*. The quality factor increases as function of the capacitance *Cc*.

(23) $$Q_{f}=R\times \sqrt{\frac{C+C_{C}}{L}}$$

## 9. Conclusions

In this paper a compact triangular triple-band antenna for RF energy harvesting applications enabling self-feeding of IoT systems is presented. Three configurations (vertical antenna (0°), tilted antennas (30°, 60° and 90°) and flexible antenna) have been studied, simulated and tested. The vertical antenna and the inclined antennas are realized on Teflon glass substrate with a thickness of 0.67 mm and a relative permittivity of 2.1. The flexible antenna is made on waterproof paper with a thickness of 0.1 mm and a relative permittivity of 2.1. The size of the antennas is of 39 × 9 mm^2^. The performance of the antenna for this size has been compared to those reported in the literature. 

The vertical antenna covers the frequencies 2.45 GHz, 5.2 GHz and 8.2 GHz with measured gains of 2.6 dBi, 4.5 dBi and 6 dBi and radiation efficiencies of 75%, 70% and 67%, respectively, for the three resonant frequencies. 

The tilted antennas are matched to the frequencies 2.45 GHz, 5.2 GHz and 8.2 GHz with negligible variation in the amplitudes of the S_11_ parameters and radiation patterns from one tilt angle to another. The maximum radiation efficiency of 75% is obtained for all tilt angles (30°, 60° and 90°) for the 2.45 GHz frequency, 72% for the 60° tilt angle for the 5.2 GHz frequency and 63% for the 30° tilted antenna for the 8.2 GHz frequency. 

The flexible antenna is adapted to the frequencies 2.45 GHz, 5.2 GHz and 8.2 GHz for an amplitude of the S_11_ parameters lower than −15 dB. The antenna on this substrate keeps the same characteristics with respect to the shape of the radiation pattern. The maximum measured gain and radiation efficiency are of 5.45 dBi and 64%, respectively. The deformation of this antenna degrades its performance slightly, such that the resonant frequencies shift to 2.65 GHz, 5.5 GHz and 8.55 GHz with relatively degraded amplitudes of the S_11_ parameter compared to those of the undeformed antenna. The maximum measured radiation efficiency and gain are, respectively, 62% and 4.7 dBi. 

By sticking the antenna to the human body, its bandwidth widens, ranging from 1.2 GHz to 10 GHz. The radiation pattern changes compared to the undeformed and deformed antenna. This change of the radiation pattern is acceptable for various applications, especially for RF energy harvesting. 

A calculation of the SAR on a 10 g tissue and an input power of 100 mW has been performed. For the three resonant frequencies, 2.45 GHz, 5.2 GHz and 8.2 GHz, the SAR is 1.1 W/kg, 0.71 W/kg and 0.45 W/kg, respectively.

All proposed antennas have been then applied to test RF energy harvesting systems for 2.45 GHz. The simulations and measurements have been done in term of S_11_ parameter of rectifiers, output DC voltage and output DC power for −20 dBm and 2 kΩ resistance load. The maximum measured output DC power is 7.68 µW with a maximum RF-to-DC conversion efficiency of 77%.

## Figures and Tables

**Table 1:** Comparison between the proposed antenna and other miniaturized antenna characteristics presented in the literature during the 10 last years.

Ref	Antenna Size (mm^3^)	Operating Frequency (GHz)	Maximum Gain (dB)	Substrate Type	Antenna Type
[11]	28.3 × 34.5 × 0.26	2.5	2.4	RO3003/Tencel	Patch antenna/CPW feed line
[12]	27 × 60 × 0.76	0.764–1.029	1.42	RO4350	Meandered loop slot-line antenna
[13]	30 × 30 × 0.24	2.45/5.8	1	Graphene	Patch antenna/CPW feed line
[14]	85 × 43 × 0.7	2.45/5.8	8.2	Multi-layers	CPW strip-line feeding
[15]	33 × 22 × 1.6	2.5/4/6	1.6	FR-4	Patch antenna
[16]	30 × 30 × 10	0.918	1	Copper	Folded dipole
[17]	31 × 18.5 × 1.6	2.15–2.9	2.2	FR-4	Fractal patch antenna
[18]	38 × 38 × 1.6	2.45	3	FR-4	Double layer of fractal patch antenna
[19]	110 × 110 × 0.1	0.875/1.94/2.6	2.3/5/4.8	Paper	Coupled proximity patches
[20]	100 × 100 × 5	2.45	8.35	FR-4	Rectangular patch antenna
[21]	110 × 60 × 0.8	0.868/0.915	2.6	FR-4	Printed dipole patch antenna
[22]	60 × 60 × 1.6	1/1.85/2.5/3.55/5.85/7.4	1/3/5/4	FR-4	Square fractal patch antenna
[23]	60 × 30 × 1.65	2.4/5.8	2/5.3	FR-4	Fractal patch antenna
[24]	70 × 70 × 0.8	2.45/5.5	3.4/6	FR-4	CPW slot antenna
[25]	76 × 46 × 1.6	2.45	2.9	FR-4	Patch antenna
[26]	120 × 65 × 1.6	5	8	FR-4	Triangular patch antenna
[27]	44 × 33 × 1.67	2.45/5.8	1.48/3.83	FR-4	Microstrip patch antenna
[28]	48 × 28 × 1.6	2.45	2.5	FR-4	Hexagonal patch antenna
This work	39 × 9 × 0.67 39 × 9 × 0.1	2.45/5.2/8.2	2.6/4.55/6 2.45/4.2/5.7	Teflon glass Waterproof paper	Triangular patch antenna

**Table 2:** Dimension values of the three patch shapes in mm.

Dimensions	W	W_Lt_	W_Lr_	W_Lc_	L_Lt_	L_Lr_	L_Lc_	L_g_	R_c_	L_t_	L_r_
Values	9	2	2	2	10	10	9.5	7	3.75	9.7	4.85

**Table 3:** Optimized antenna dimensions in (mm).

Dimensions	W_t_	L_t_	S	F	g	W
Values	9	39	10.66	10	2	2

**Table 4:** Values of Δf and Q for the three resonant frequencies.

f_r_ (GHz)	2.45	5.2	8.15
∆f (MHz)	6.6	20	32.16
Q	378	246.7	248.7

**Table 5:** Values of R, L and C of each resonator of the electrical equivalent circuit.

Elements	R_1_ (Ω)	R_2_ (Ω)	R_3_ (Ω)	L_1_ (nH)	L_2_ (nH)	L_3_ (nH)	C_1_ (pF)	C_2_ (pF)	C_3_ (pF)
Values	49.5	46.2	49.5	0.1	0.17	0.49	3.92	5.95	8.4

**Table 6:** LC elements Values of the impedance matching circuit for both used substrates.

	Elements	L_1_ (nH)	L_2_ (nH)	C_1_ (pF)	C_2_ (pF)
Substrates					
Teflon glass		10	0.7	6.45	30
Waterproof paper		9.9	0.62	8.8	21

**Table 7:** Parasitic elements values of the impedance matching circuit for both used substrates.

	Elements	L_S1_ (pH)	L_S2_ (pH)	R_S1_ (mΩ)	R_S2_ (mΩ)	R_LS1_ (mΩ)	R_LS2_ (mΩ)	C_L1_ (pF)	C_L2_ (pF)
Substrates									
Teflon glass		10	7	10	50	80	10	0.02	0.01
Waterproof paper		5	10	10	42	50	12	0.03	0.001

**Table 8:** Measured output DC voltages, output DC powers and RF-to-DC conversion efficiencies of the three RF-EH system configurations (using the tilted antennas of 0°, 30°, 60° and 90°) realized on Teflon glass substrate for −20 dBm input power and 2 kΩ load.

RF-EH Systems	0°	30°	60°	90°
Output DC voltage (mV)	112	103	108	110
Output DC power (µW)	6.27	5.3	5.8	6
RF-to-DC efficiency (%)	63	53	58.3	60.5

**Table 9:** Measured output DC voltages, output DC powers and RF-to-DC conversion efficiencies of the RF-EH system realized on WP substrate for −20 dBm input power and 2 kΩ load.

RF-EH Systems	Undeformed	Deformed	On Human Body
Output DC voltage (mV)	119	110	124
Output DC power (µW)	7.1	6	7.68
RF-to-DC efficiency (%)	70.8	60.5	77

**Table 10:** Comparison between the proposed RF-EH system and other miniaturized systems characteristics presented in the literature.

Ref	System Size (mm^3^)	Operating Frequency (GHz)	Output DC Power (µW)	RF-to-DC Conversion Efficiency (%)	Substrate Type
[27]	80 × 48 × 1.67	2.45	160 @ 2 dBm	45 @ +2 dBm	FR-4
[43]	70 × 87 × 3.63	2.45	3.36 @ −20 dBm	33.6 @ −20 dBm	Woven polyester and polyester felt
[48]	200 × 150 × 2.8	2.45	80 @ 16 mW/m^2^	36.4 @ 16 mW/m^2^	double-layer PTFE
[49]	150 × 80 × 4	2.45	3.1 @ −20 dBm	31 @ −20 dBm	Rogers 5880
[50]	45 × 45 × 0.8	1.81	46.9 @ −9.6 dBm	61 @ −3.1 dBm	2 layers PCB
[51]	100 × 390 × 0.9	1.8 + 2.15	4 @ −20 dBm	40 @ −20 dBm	RT/Duroid 5880
[52]	60 × 60 × 0.76	0.915/2.45	4.55 @ −15 dBm	20 @ −15 dBm	Arlon 25N
[53]	100 × 70 × 40	2.45	6.5 @ 0.2 mW/m^2^	74 @ 0.2 mW/m^2^	FR-4
[54]	70 × 70 × 6	2.45	79 @ 50 mW/m^2^	64 @ 295 mW/m^2^	RO4350B
[55]	78 × 135 × 1.6	2.1	15 @ −10 dBm	15 @ −10 dBm	FR-4
[56]	100 × 100 × 70	2.45	9.7 @ −10 dBm	35 @ +10 dBm	FR-4
[57]	175 × 200 × 84	0.9 + 1.8 + 2.17	162 @ 1 mW/m^2^	35 @ −20 dBm	RT/Duroid 5880
This work	54 × 20 × 0.67 59 × 20 × 0.1	2.45	6.27 @ −20 dBm 7.8 @ −20 dBm	63 @ −20 dBm77 @ −20 dBm	Teflon glass Waterproof paper

**Table 11:** Values of the coupling capacitances of each tilt.

Angles	30°	60°	90°
*F_r_*_,__*sim*_ (GHz)	8.4	8.4	8.4
*F_r_*_,__*meas*_ (GHz)	8.2	8.1	8
*C_C_* (pF)	0.24	0.346	0.45

## Data Availability

Not applicable.
